# Group vs Individual Prenatal Care and Gestational Diabetes Outcomes: A Secondary Analysis of a Randomized Clinical Trial

**DOI:** 10.1001/jamanetworkopen.2023.30763

**Published:** 2023-08-29

**Authors:** Yixin Chen, Amy H. Crockett, Jessica L. Britt, Lu Zhang, Roch A. Nianogo, Tianchen Qian, Bin Nan, Liwei Chen

**Affiliations:** 1 Department of Epidemiology, Fielding School of Public Health, University of California, Los Angeles; 2 Division of Maternal Fetal Medicine, Department of Obstetrics and Gynecology, Prisma Health, Greenville, South Carolina; 3 University of South Carolina School of Medicine, Greenville; 4 Department of Obstetrics and Gynecology, Prisma Health, Greenville, South Carolina; 5 Department of Public Health Sciences, Clemson University, Clemson, South Carolina; 6 California Center for Population Research, Los Angeles; 7 Department of Statistics, University of California, Irvine

## Abstract

**Importance:**

The impact of group-based prenatal care (GPNC) model in the US on the risk of gestational diabetes (GD) and related adverse obstetric outcomes is unknown.

**Objective:**

To determine the effects of the GPNC model on risk of GD, its progression, and related adverse obstetric outcomes.

**Design, Setting, and Participants:**

This is a single-site, parallel-group, randomized clinical trial conducted between February 2016 and March 2020 at a large health care system in Greenville, South Carolina. Participants were individuals aged 14 to 45 years with pregnancies earlier than 21 weeks’ gestational age; follow-up continued to 8 weeks post partum. This study used an intention-to-treat analysis, and data were analyzed from March 2021 to July 2022.

**Interventions:**

Eligible participants were randomized to receive either CenteringPregnancy, a widely used GPNC model, with 10 group-based sessions or traditional individual prenatal care (IPNC).

**Main Outcomes and Measures:**

The primary outcome was the incidence of GD diagnosed between 24 and 30 weeks of gestation. The secondary outcomes included progression to A2 GD (ie, GD treated with medications) and GD-related adverse obstetric outcomes (ie, preeclampsia, cesarean delivery, and large for gestational age). Log binomial models were performed to estimate risk differences (RDs), 95% CIs, and *P* values between GPNC and IPNC groups, adjusting for all baseline covariates.

**Results:**

Of all 2348 participants (mean [SD] age, 25.1 [5.4] years; 952 Black participants [40.5%]; 502 Hispanic participants [21.4%]; 863 White participants [36.8%]), 1176 participants were randomized to the GPNC group and 1174 were randomized to the IPNC group. Among all participants, 2144 (91.3%) completed a GD screening (1072 participants [91.3%] in GPNC vs 1071 [91.2%] in IPNC). Overall, 157 participants (6.7%) developed GD, and there was no difference in GD incidence between the GPNC (83 participants [7.1%]) and IPNC (74 participants [6.3%]) groups, with an adjusted RD of 0.7% (95% CI, −1.2% to 2.7%). Among participants with GD, GPNC did not reduce the risk of progression to A2 GD (adjusted RD, −6.1%; 95% CI, −21.3% to 9.1%), preeclampsia (adjusted RD, −7.9%; 95% CI, −17.8% to 1.9%), cesarean delivery (adjusted RD, −8.2%; 95% CI, −12.2% to 13.9%), and large for gestational age (adjusted RD, −1.2%; 95% CI, −6.1% to 3.8%) compared with IPNC.

**Conclusions and Relevance:**

In this secondary analysis of a randomized clinical trial among medically low-risk pregnant individuals, the risk of GD was similar between participants who received GPNC intervention and traditional IPNC, indicating that GPNC may be a feasible treatment option for some patients.

**Trial Registration:**

ClinicalTrials.gov Identifier: NCT02640638

## Key Points

**Question:**

Does receiving group prenatal care vs individual prenatal care reduce the risk of gestational diabetes (GD)?

**Findings:**

In this large randomized clinical trial including 2348 participants, pregnant individuals receiving group prenatal care had a similar risk of developing GD as those receiving traditional individual prenatal care.

**Meaning:**

These findings suggest that individuals receiving both group prenatal care and traditional individual prenatal care had similar risk of developing GD, indicating that group prenatal care may be a possible treatment option for some individuals.

## Teaser

This secondary analysis of a randomized clinical trial compares group prenatal care vs traditional individual prenatal care for the prevention of gestational diabetes and related adverse obstetric outcomes.

## Introduction

Gestational diabetes (GD) is 1 of the most common pregnancy complications, affecting 4% to 8% of all pregnancies depending on diagnostic criteria and study populations.^1,2^ In the US, the incidence of GD has increased more than 10-fold in the last 40 years.^1,3^ It is well-documented that the burden of GD differs by race and ethnicity.^4^ In the US, the prevalence of GD has been found to be highest among Asian individuals, followed by Hispanic, Black, and White individuals.^5^ Moreover, pregnancies affected by GD have increased risks of adverse outcomes, such as preeclampsia, primary cesarean delivery, birth injury, macrosomia, and large-for-gestational-age (LGA) birth.^4,6^ With its high prevalence and potential for adverse impact on both maternal and offspring health, prevention and management of GD is a major challenge for obstetrical care practitioners.

Lifestyle interventions (ie, diet and physical activity) have achieved a modest effect on improving rates of GD.^7,8,9^ Among the existing studies, including those with high quality and large sample sizes, very few include racially and ethnically diverse study populations. Strategies to enhance psychosocial health have been shown to reduce anxiety, empower individuals, and result in better glycemic control among individuals with GD,^10,11,12^ but they are not aimed to reduce GD risk. Group prenatal care (GPNC) is an innovative model of medical care to improve clinical outcomes and health behaviors and to reduce racial disparities.^13,14,15,16,17,18^ CenteringPregnancy (Centering Healthcare Institute, Boston, Massachusetts) is the most widely implemented GPNC model and includes a curriculum specifically targeting elements of healthy lifestyle and behaviors (eg, nutrition, exercise, goal setting, and self-care) and psychosocial improvement (eg, stress management, empowerment, and peer and family support).^19,20^ Previous studies^21,22,23,24,25,26,27,28,29,30,31^ have found that pregnant participants receiving GPNC had less excessive gestational weight gain and better attendance at postpartum visits compared with those receiving traditional individual prenatal care (IPNC). The literature^31,32,33,34^ also includes reports of lower risk of GD, reduced progression to A2 GD (indicated by medication for glucose control during pregnancy), and lower fasting plasma glucose levels for patients receiving GPNC compared with IPNC. In this secondary analysis of the CRADLE (Centering and Racial Disparities) study,^15,35^ we aimed to determine whether GPNC, compared with IPNC, leads to lower risks of GD, progression to A2 GD, and GD-related adverse obstetric outcomes in a large randomized clinical trial (RCT).

## Methods

### Study Design and Participants

This study is a secondary analysis of the CRADLE study,^15,35^ a single-site RCT among racially diverse pregnant participants designed to evaluate the effects of GPNC on preterm birth and low birth weight. Evaluating the effect of GPNC on incidence and progression of GD was a prespecified secondary outcome.^35^ This study was conducted in accordance with the Consolidated Standards of Reporting Trials (CONSORT) reporting guideline,^36^ and was approved by the institutional review boards of Prisma Health and the University of South Carolina School of Medicine-Greenville. Eligible participants in the CRADLE study provided written informed consent. The study design has been previously published,^35^ and the full study protocol is included in Supplement 1. The Figure and eFigure in Supplement 2 show the CONSORT flow diagrams for our intent-to-treat (ITT) and modified ITT (mITT) samples, respectively.^36^

**Figure.**
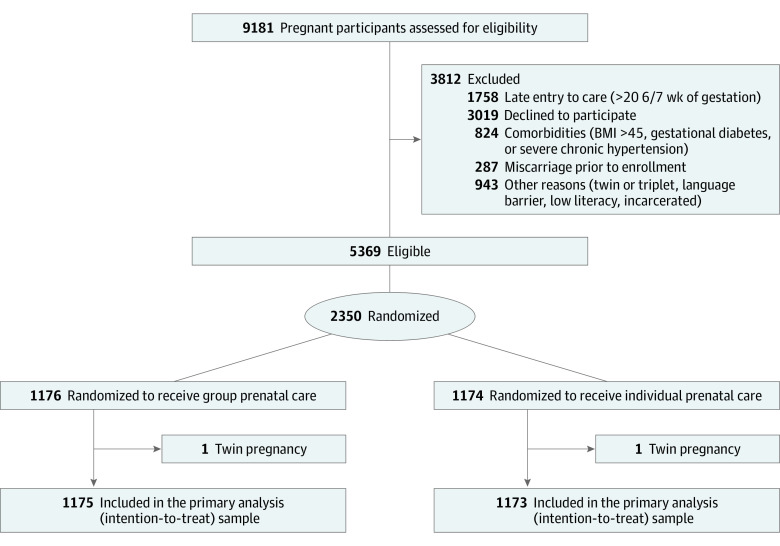
Centering and Racial Disparities Trial Diagram

Enrollment for the CRADLE study began on February 24, 2016, and ended on March 16, 2020, when our capacity for safe in-person GPNC sessions was limited by the COVID-19 pandemic.^15,35^ The eligibility criteria included singleton pregnancy, age 14 to 45 years, and entry to prenatal care prior to 20 weeks and 6 of 7 days of gestation. Participants were excluded if they had prepregnancy diabetes, a body mass index (calculated as weight in kilograms divided by height in meters squared) of 45 or greater, chronic hypertension, or other medical or psychosocial complications that would make them ineligible to receive care in a group setting or from nurse practitioners (eg, active tuberculosis, current incarceration, or severe uncontrolled psychiatric illness). The exclusion criteria aimed to limit the study participants to low-risk pregnant participants, which was consistent with the GPNC model design. Eligible participants were followed up through pregnancy until 8 weeks post partum.

### Randomization and Blinding

Eligible participants were randomly assigned in a 1:1 allocation, stratified by race and ethnicity, to receive either GPNC (intervention) or IPNC (control) as their prenatal care. Due to the nature of the intervention, participants and practitioners were not blinded to group assignments. However, the study analysts were blinded to group assignments to reduce bias.

### Interventions

Participants in the IPNC group (control) received traditional prenatal care (an average 15 minutes for each visit) following the schedule of prenatal care visits recommended by the American College of Obstetricians and Gynecologists, which typically recommends a total of 13 visits for uncomplicated pregnancies.^37^ Participants in the GPNC group (intervention) were organized into groups of 8 to 12 patients due to deliver in approximately the same month and scheduled to attend 10 2-hour group sessions following the standard curriculum provided by Centering Healthcare Institute.^19^ GPNC participants were allowed to have additional IPNC visits (in addition to the 10 scheduled group sessions) as needed. The study site was able to deliver both interventions in English and Spanish.

### Data Collection and Measures

Over the course of the study, 2 surveys were administered: survey 1 at baseline (<24 gestational weeks) and survey 2 between 30 and 36 gestational weeks. Both surveys were designed and administered using REDCap (Research Electronic Data Capture) software version 11.4.4 and delivered via tablet computers (iPad; Apple) during clinic visits.^38^ The surveys covered demographic questions including detailed information about self-reported race and ethnicity, psychosocial measures, maternal health behavior, lifestyle measures, and reproductive and medical history.^35^ By using REDCap, we allowed participants to privately self-identify race and ethnicity with more specificity, using validated questions aligned with US Federal Government standards from 1997. These questions enabled participants to choose multiple race and ethnicity categories and provide open-ended descriptions, ensuring a comprehensive and detailed report of their racial and ethnic background.^39^ In the analysis, race and ethnicity categories included Black, Hispanic, White, and other (defined as Asian, Native Hawaiian, Other Pacific Islander, or unknown) or multiracial (categorized in the study questionnaire as *mixed race*). All study participants were biologically females. At 8 weeks after delivery, participants’ medical information was abstracted from the electronic medical record by trained research personnel using a prespecified data abstraction form.

### Primary and Secondary Outcomes

Incidence of GD was the primary outcome for this study and was diagnosed according to the American College of Obstetricians and Gynecologists recommendation using 2-step screening, which was the standard practice at the clinical site.^40^ Participants were screened for GD between 24 and 30 weeks of gestation with the 50-g oral glucose challenge test. Results greater than 200 mg/dL (to convert glucose to millimoles per liter, multiply by 0.0555) were diagnosed as GD. Participants with oral glucose challenge test results between 140 and 200 mg/dL took a 100-g, 3-hour oral glucose tolerance test. Those with 2 elevated values meeting the Carpenter and Coustan^40^ criteria (ie, fasting ≥95 mg/dL, 1-hour ≥180 mg/dL, 2-hour ≥155 mg/dL, or 3-hour ≥140 mg/dL) received a diagnosis of GD.

Secondary outcomes included progression from the White classification^40^ A1 GD to A2 GD and adverse obstetric outcomes. All participants with a diagnosis of GD were initially categorized as class A1 and were prescribed nutritional therapy. If participants were unable to maintain more than 70% to 80% of blood glucose levels within the target range through nutritional therapy, medications were recommended, and they would be reclassified as A2 GD. White classification^40^ A2 indicates a more severe level of glucose intolerance, posing additional risks for adverse obstetric outcomes. Twice-weekly antenatal testing is recommended and scheduled until delivery at 39 weeks of gestation for these patients to minimize fetal risk.^40^

We evaluated adverse obstetric outcomes associated with poor glycemic control individually. These outcomes included primary cesarean delivery, preeclampsia (ie, onset at >20 gestational weeks, 24-hour proteinuria ≥30 mg/day, a protein concentration ≥30 mg in at least 2 random urine samples, or a systolic/diastolic blood pressure ≥140 mm Hg/≥90 mm Hg),^41^ and LGA (ie, birth weight ≥90th percentile according to a fetal growth curve).^42,43^

### Statistical Analysis

The primary analyses compared the primary and secondary outcomes between GPNC and IPNC groups using the ITT approach. The ITT sample included all study participants according to their randomized assignment, regardless of missed visits, crossover between IPNC and GPNC, or transfer out of the practice. In the sensitivity analyses, a mITT approach was used, excluding participants with (1) spontaneous abortion, (2) intrauterine fetal demise, (3) incomplete information on the number of prenatal care visits, and (4) no postrandomization prenatal care visits in their assigned study group. According to our previously published observational study,^31^ assuming the rate of GD was 4.1% in the GPNC group and 6.4% in the IPNC group, the post hoc power of this study is 70%, with an α of .05.

For the description of baseline characteristics and prognostic factors, continuous variables were described by mean (SD), and categorical variables were described by count (percentage). The missingness of the baseline characteristics and prognostic factors were mainly due to participants’ preference of not answering. The missingness of marital status (516 participants [22.0%]), annual household income (714 participants [30.4%]), and health insurance (270 participants [11.5%]) were greater than 10% and were kept as a separate category during the statistical analysis. Missing data of other variables were lower than 7%, ranging from 2 participants [0.1%] for prepregnancy BMI to 63 participants [6.9%] for GWG. Log binomial models were performed to estimate risk differences (RDs), 95% CIs, and *P* values between GPNC and IPNC groups, adjusting for all baseline covariates. Stratified analyses were performed to estimate RD of GD by racial and ethnic categories according to the study design.^35^ A sensitivity analysis was also conducted by excluding participants whose study participation could be affected by COVID-19. All analyses were performed using SAS statistical software version 9.4 (SAS Institute), and a 2-sided *P* < .05 was considered statistically significant. Data analysis was conducted from March 2021 to July 2022.

## Results

Among 2350 enrolled participants (1176 participants randomized to the GPNC group and 1174 randomized to the IPNC group), 2 twin pregnancies (1 in GPNC and 1 in IPNC) were identified after randomization and excluded, given the study eligibility criteria, resulting in a total of 2348 participants (mean [SD] age 25.1 [5.4] years] with 1175 participants in the GPNC group and 1173 in the IPNC group in the ITT sample (Figure). Among them, 2144 (91.3%) had completed a screening for GD (1072 participants in the GPNC group and 1071 in the IPNC group). All participants with GD had information on GD progression (83 participants in the GPNC group and 74 in the IPNC group) and 93.6% (76 participants in the GPNC group and 71 in the IPNC group) had data regarding delivery and birth outcomes.

### Baseline and Prognostic Characteristics

The study population was racially diverse, with 952 Black participants (40.5%), 502 Hispanic participants (21.4%), 863 White participants (36.8%), and 31 participants (1.3%) who identified as other race or multiracial. Baseline characteristics and prognostic factors were similar between the 2 groups (Table 1), except smoking 3 months before pregnancy (454 participants [38.7%] in the IPNC group vs 386 participants [32.9%] in the GPNC group).

**Table 1.** Baseline Characteristics and Associated Factors for Gestational Diabetes in the Centering and Racial Disparities Study^a^

Characteristic	Participants, No. (%) (N = 2348)	
	Individual prenatal care (n = 1173)	Group prenatal care (n = 1175)
Race and ethnicity		
Black	476 (40.6)	476 (40.5)
Hispanic	249 (21.2)	253 (21.5)
White	433 (36.9)	430 (36.6)
Other or multiracial^b^	15 (1.3)	16 (1.4)
Maternal age, mean (SD), y	25.0 (5.3)	25.3 (5.4)
Prepregnancy body mass index, mean (SD)^c^	28.8 (7.2)	29.0 (7.2)
Prepregnancy body mass index status^c^		
Underweight (<18.5)	39 (3.3)	39 (3.3)
Normal weight (18.5 to <25.0)	391 (33.3)	370 (31.5)
Overweight (25.0 to <30.0)	297 (25.3)	292 (24.9)
Obese (≥30.0)	446 (38.0)	474 (40.3)
High school education or above	819 (69.8)	830 (70.6)
Student status for the past year		
Not a student	825 (70.3)	835 (71.1)
High school student (or working on general education development)	151 (12.9)	130 (11.1)
Community college, technical college, and 4-y college students	143 (12.2)	134 (11.4)
Employment		
Employed	600 (51.2)	601 (51.2)
Unemployed	257 (21.9)	265 (22.6)
Keeping house or caring for family full time	251 (21.4)	239 (20.3)
Marital status		
Married	704 (60.0)	662 (56.3)
Unknown	230 (19.6)	286 (24.3)
Annual household income, $		
<10 000	252 (21.5)	282 (24.0)
10 000 to <20 000	236 (20.1)	233 (19.8)
20 000 to <50 000	293 (25.0)	273 (23.2)
≥50 000	34 (2.9)	31 (2.6)
Unknown	358 (30.5)	356 (30.3)
Health insurance		
Had health insurance in the past year	579 (49.4)	563 (47.9)
Unknown	137 (11.7)	133 (11.3)
Nulliparous	522 (44.5)	522 (44.4)
Perceived family support score, mean (SD)	3.4 (0.6)	3.4 (0.6)
Smoked tobacco during the 3 mo before pregnancy	454 (38.7)	386 (32.9)
Pregnancy		
Unintended	753 (64.2)	749 (63.7)
Unknown	52 (4.4)	40 (3.4)
Prognostic factors		
Total gestational weight gain, mean (SD), lb	24.5 (16.6)	24.1 (16.1)
Smoked tobacco during the pregnancy	220 (18.8)	196 (16.7)
Consumed alcohol during the pregnancy	43 (3.7)	52 (4.4)
Smoked marijuana during the pregnancy	54 (4.6)	60 (5.1)

SI conversion factor: To convert pounds to kilograms, multiply by 0.45.

^a^ The analyses were conducted among the intention-to-treat sample.

^b^ Other indicated Asian, Native Hawaiian, Other Pacific Islander, or unknown race.

^c^ Prepregnancy body mass index was measured at first prenatal care visit and was calculated as weight in kilograms divided by height in meters squared.

### Primary Outcome: GD Incidence

Overall, 157 (6.7%) participants developed GD, and there was no difference between incidence of GD in the GPNC group (83 participants [7.1%]) and IPNC group (74 participants [6.3%]) groups (adjusted RD, 0.7%; 95% CI, −1.2% to 2.7%) (Table 2). In the subgroup analysis, the RDs of GD comparing GPNC with IPNC did not vary across participants of different races and ethnicities. The adjusted RD was 0.5% (95% CI, −2.0% to 3.0%) for Black participants, −0.5% (95% CI, −5.8% to 4.9%) for Hispanic participants, and 2.6% (95% CI, −0.7% to 6.0%) for White participants (Table 2).

**Table 2.** Comparing the Risk of Gestational Diabetes by Intervention Type in the Centering and Racial Disparities Study^a^

Participant race or ethnicity	Incidence of gestational diabetes, No. of participants/total No. (%) (N = 2438)		Risk difference (95% CI)			
	Individual prenatal care (n = 1173)	Group prenatal care (n = 1175)	Unadjusted	*P* value	Adjusted^b^	*P* value
All participants	74/1173 (6.3)	83/1175 (7.1)	0.8 (−1.3 to 2.8)	.46	0.7 (−1.2 to 2.7)	.46
Black participants	19/476 (4.0)	22/476 (4.6)	0.6 (−2.0 to 3.2)	.63	0.5 (−2.0 to 3.0)	.70
Hispanic participants	27/249 (10.8)	24/253 (9.5)	−0.5 (−6.4 to 5.5)	.88	−0.5 (−5.8 to 4.9)	.86
White participants	25/433 (5.8)	36/430 (8.4)	2.6 (−0.8 to 6.0)	.14	2.6 (−0.7 to 6.0)	.12

^a^ The analyses were conducted among the intention-to-treat sample.

^b^ The regression model was adjusted for all baseline characteristics including smoking before pregnancy, race and ethnicity, maternal age, body mass index (calculated as weight in kilograms divided by height in meters squared) at enrollment, education, employment, student status for the past year, annual household income, had dental visit within the last 2 years, had insurance for the past year, parity, marital status, and unintended pregnancy.

### Secondary Outcomes

Overall, 49.0% of participants (76 of 157 participants) with GD progressed to A2 GD. The incidence of A2 GD was 48.2% (39 of 83 participants) in the GPNC group and 50.0% (37 of 74 participants) in the IPNC group. The adjusted RD was −6.1% (95% CI, −21.3% to 9.1%) after controlling for all baseline characteristics (Table 3). The proportions of preeclampsia (7.2% [6 participants] in the GPNC group vs 16.2% [7 participants] in the IPNC group), primary cesarean delivery (10.8% [9 participants] in the GPNC group vs 16.2% [12 participants] in the IPNC group), and LGA (2.4% [2 participants] in the GPNC group vs 4.1% [3 participants] in the IPNC group) were slightly lower in the GPNC group, but the differences were not statistically significant (Table 3). Comparing GPNC with IPNC controlling for all baseline characteristics, the adjusted RD was −7.9% (95% CI, −17.8% to 1.9%) for preeclampsia, −8.2% (95% CI, −12.2% to 13.9%) for cesarean delivery, and −1.2% (95% CI, −6.1% to 3.8%) for LGA.

**Table 3.** Comparing the Risks of Progression to A2 GD and GD–Related Obstetric Outcomes by Intervention Type in the Centering and Racial Disparities Study^a^

Outcome	Participants with GD, No. (%) (N = 157)		Risk difference (95% CI)			
	Individual prenatal care (n = 74)	Group prenatal care (n = 83)	Unadjusted	*P* value	Adjusted^b^	*P* value
Progression to A2 GD^c^	37 (50.0)	39 (48.2)	−1.9 (−17.6 to 13.9)	.82	−6.1 (−21.3 to 9.1)	.51
Preeclampsia	12 (16.2)	6 (7.2)	−9.0 (−19.1 to 1.1)	.09	−7.9 (−17.8 to 1.9)	.12
Cesarean delivery	12 (16.2)	9 (10.8)	−1.4 (−14.7 to 11.9)	.83	−8.2 (−12.2 to 13.9)	.90
Large for gestational age	3 (4.1)	2 (2.4)	−1.6 (−7.2 to 3.9)	.66	−1.2 (−6.1 to 3.8)	.66

Abbreviations: GD, gestational diabetes.

^a^ The analyses were conducted among the intention-to-treat sample.

^b^ The regression model was adjusted for all baseline characteristics including smoking before pregnancy, race and ethnicity, maternal age, body mass index (calculated as weight in kilograms divided by height in meters squared) at enrollment, education, employment, student status for the past year, annual household income, had a dental visit within the last 2 years, had insurance for the past year, parity, marital status, and unintended pregnancy.

^c^ A2 GD refers to GD managed with medication.

### Sensitivity Analyses

For sensitivity analyses, 825 participants in the GPNC group and 1099 in the IPNC group were left in the mITT sample (eFigure in Supplement 2). Baseline characteristics and prognostic factors for GD were similar between the 2 groups, except for smoking 3 months before pregnancy (eTable 1 in Supplement 2). In general, the results from the mITT sample were similar to those of the ITT sample (eTable 2 and eTable 3 in Supplement 2).

We tracked 171 participants (85 in GPNC and 86 in IPNC) who were enrolled before March 17, 2020, whose study participation could have been influenced by the pandemic. Excluding these 171 participants yielded similar results (eTable 4 and eTable 5 in Supplement 2).

## Discussion

In this secondary analysis of a large RCT, among 2348 pregnant participants from a single health care system, we found the risks of GD, progression to A2 GD, and GD-related adverse obstetric outcomes were similar between GPNC and IPNC groups. We did not find that the effect of GPNC on the risk of developing GD was different across participants of different races and ethnicities.

Our RCT design is an important contribution to the understanding of the impact of GPNC on the risk of developing GD. Our team previously reported that participants receiving the GPNC had 42% lower odds of developing GD (odds ratio, 0.58; 95% CI, 0.38-0.89), compared with participants receiving IPNC in a large retrospective cohort of racially diverse pregnant participants.^31^ However, the CRADLE study found similar risk of GD between GPNC and IPNC groups. A possible explanation is residual confounding in the previous observational study (ie, GPNC participants could have different unmeasured characteristics compared with IPNC participants). In addition, participants in the observational study self-selected to receive GPNC, whereas participants were randomly assigned to GPNC in the CRADLE RCT. Low attendance was observed in the GPNC group, with only one-half of the participants attending 5 or more group sessions, 3.9% attending all 10 sessions, and a significant portion not attending any group sessions (25%).^15^ Our previous results found not liking GPNC (16.43%) was the primary reason for low attendance in the CRADLE RCT, but this was unlikely a problem for observational studies since the participants were allowed to select either GPNC or IPNC by their wishes.^44,45^ Poor adherence to the assigned intervention can attenuate the intervention effects in a RCT.^46,47,48,49^ Also, the requirement of twice-weekly prenatal visits after 34 weeks of gestation for participants with A2 GD in the GPNC group may have impacted their attendance. Nevertheless, poor adherence to GPNC group session remained a problem. Of course, we cannot rule out the possibility that GPNC has a similar effect on GD onset or progression compared with IPNC.

The diverse study population allowed us to explore potential variations in the benefits of GPNC among participants of different races and ethnicities. In our previous observational study,^50^ we observed Black pregnant participants receiving GPNC had a greater reduction in the risk of preterm birth compared with White participants. However, in the CRADLE study, we did not find a difference in the risk of GD across participants of different races and ethnicities when comparing GPNC with IPNC. Again, the inconsistent results between the observational study and RCT could be due to the biases arising from each study design, but it highlighted the need of future studies to explore whether pregnant people, especially pregnant people of different races or ethnicities, may benefit more from GPNC prenatal care.

A few observational studies^32,33^ compared the association of GPNC and IPNC progression with A2 GD among participants with GD. Comparing 62 participants with GD receiving GPNC with 103 participants receiving IPNC, Mazzoni et al^33^ reported an odds ratio of 0.15 for progression to A2 GD (95% CI, 0.07-0.30). A lower risk of progression to A2 GD was also reported among Hispanic participants with GD (203 receiving GPNC and 257 receiving IPNC).^32^ Our results, despite being only suggestive, are in line with the findings from previous observational studies. Taken together, these data call for future studies to test the association of GPNC with GD progression, glycemic control, and GD-related obstetric and neonatal outcomes among people with GD.

A unique strength of this study is the eligibility criteria were consistent with what practitioners generally apply when recruiting for GPNC and therefore reflected the typical clinical context and maximized the generalizability of the study findings. Our study site has extensive experience in offering CenteringPregnancy GPNC for pregnant participants (ie, the site has been continuously certified by the Centering Healthcare Institute since 2009), indicating a high degree of fidelity to the model. In the current study, the GPNC was provided in both English and Spanish. Additional strengths of our study include the large sample size (>2000 participants), the racially and ethnically diverse population, and the rich measures of sociodemographic, psychosocial, behavioral, reproductive, and clinical factors at baseline and over pregnancy.

### Limitations

Our study had several limitations. First, the early termination of the trial due to the COVID-19 pandemic resulted in a smaller sample size than originally planned, limiting the power for our secondary analysis. According to our previously published observational study,^31^ assuming the rate of GD was 4.1% in the GPNC group and 6.4% in the IPNC group, the post hoc power of this study is 70%, with an α of .05. The power was even more limited to detect GD progression and related adverse obstetric outcomes and to assess racial and ethnic disparities in the risks of our secondary outcomes. Second, we observed low attendance among GPNC participants, despite implementing strategies such as offering onsite professional childcare during group sessions and transportation reimbursement, which are often unavailable in practice settings outside of a research context. Still the attendance at the group sessions was lower than expected. Furthermore, our findings may be generalized to medically low-risk pregnant people only. However, considering GPNC model curriculum covers multiple modifiable factors (ie, psychosocial well-being, peer and family support, and healthy diet) associated with the lower risk of GD and other pregnancy complications, it could be an option for pregnant women seeking prenatal care.

## Conclusions

In this RCT among pregnant individuals, participants receiving GPNC had similar risk of developing GD, compared with participants receiving IPNC, suggesting that GPNC could be a feasible care option for some patients. Future studies should explore additional strategies to enhance participant engagement with GPNC. Moreover, research is needed to assess the effect of GPNC on GD progression among pregnant individuals with GD.

## Notes

Supplement 1.
Trial Protocol and Statistical Analysis Plan

Supplement 2. **eFigure.** CRADLE Trial CONSORT Diagram Showing Eligibility, Randomization, Follow-Up, and Outcomes Availability, Modified Intent-to-Treat (MITT) Sample **eTable 1.** Baseline Characteristics and Prognostic Risk Factors for GDM by Intervention Arms: Group Prenatal Care (GPNC) Group vs. Individual Prenatal Care (IPNC) Group in CRADLE Study **eTable 2.** Modified Intention-to-Treat Analysis for Comparing the Risk Of GDM by Intervention Arms: Group Prenatal Care (GPNC) Group vs. Individual Prenatal Care (IPNC) Group in CRADLE Study **eTable 3.** Modified Intention-to-Treat Analysis for Comparing the Risks of Progression to A2 GDM and GDM-Related Obstetric Outcomes by Intervention Arms: Group Prenatal Care (GPNC) Group vs. Individual Prenatal Care (IPNC) Group in CRADLE Study **eTable 4.** Sensitivity Analysis for Comparing the Risk of GDM by Intervention Arms: Group Prenatal Care (GPNC) Group vs. Individual Prenatal Care (IPNC) Group in CRADLE Study **eTable 5.** Sensitivity Analysis for Comparing the Risks of Progression to A2 GDM and GDM-Related Obstetric Outcomes by Intervention Arms: Group Prenatal Care (GPNC) Group vs. Individual Prenatal Care (IPNC) Group in CRADLE Study

Supplement 3.
Data Sharing Statement
