# Supplementary material for: Group vs Individual Prenatal Care and Gestational Diabetes Outcomes: A Secondary Analysis of a Randomized Clinical Trial
Source: JAMA Netw Open. 2023 Aug 29;6(8):e2330763. doi: 10.1001/jamanetworkopen.2023.30763 (PMC10466168; doi:10.1001/jamanetworkopen.2023.30763)

Dr. Amy H. Picklesimer, MD
Dr. Liwei Chen, PhD, MD
Dr. Xiaoqian Sun, PhD
Dr. Emily Heberlein, PhD
Sarah Covington-Kolb, MPH, MSW

A. Objective

Specific Aim 1: To compare the rate of preterm birth (PTB) prior to 37 weeks gestational age and other selected birth outcomes and pregnancy complications (e.g. birth weight, caesarean section rate) among women who participate in CenteringPregnancy group prenatal care (GPNC) to their counterparts in individual prenatal care (IPNC)

> <u>Study Hypothesis 1:</u> *Women who participate in GPNC will have a lower rate of PTB and other improved birth outcomes as compared to their counterparts in IPNC.*

Specific Aim 2: To compare the risk difference of PTB and other selected birth outcomes of Black women vs. White women in GPNC to the risk differences of Black women vs. White women in IPNC.

> <u>Study Hypothesis 2:</u> *The risk difference of PTB and other selected birth outcomes between Black and White women in the GPNC group is smaller than that in the IPNC group.*

Specific Aim 3: To compare the whether women in the GPNC have improved maternal psychosocial (i.e. activation, engagement, stress) and behavioral (i.e. smoking, healthy eating, healthy practices) outcomes as compared to their counterparts in IPNC and to explore whether improving certain maternal psychosocial and behavioral outcomes will explain the potential benefits of the GPNC on racial disparities in birth outcomes.

B. Background

Clinical interventions to reduce PTB and low birth weight (LBW) are in the highest tier of priority topics for the Institute of Medicine (IOM) research agenda (IOM, 2009). It is well known that PTB occurs at unacceptably high rates within the United States (U.S.), in which 11.5% of babies are born at less than 37 weeks gestational age. Large racial disparities persist, particularly between White and Black women; the latter had a 16.5% PTB rate and 13.2% LBW rate in 2012. PTB is the leading cause of newborn death and disability. Moreover, the racial disparities in the newborn period have the potential to contribute to disparities in chronic disease, and academic and economic achievement across the lifespan. **To date, medical and public health interventions have achieved limited success in improving rates of PTB.** The causes of poor birth outcomes and health disparities are complex, involving biological, behavioral, psychosocial, socio-demographic, environmental, and medical factors. **Interventions that do not address these factors in a comprehensive way will have limited success. Innovative models in prenatal care are therefore highly desired**.

Studies of CenteringPregnancy group prenatal care (GPNC), where individual physical assessments are combined with facilitated group education and support, have established some promising results, including high rates of prenatal care use and care satisfaction, improvements in PTB rates, particularly among Black women, and improvements in some psychosocial outcomes among women experiencing high levels of prenatal stress (Ickovics et al., 2007, 2011; Novick et al., 2011; Picklesimer et al., 2012). The body of literature evaluating birth outcomes for women participating in GPNC although growing, is still small. **Most studies in the literature are observational studies that are limited by self-selection bias and potential confounding.** Moreover, **no clinical trials have been adequately powered to conclusively determine the effects of GPNC on PTB or on racial disparities or birth outcomes.** Prior research has also not established GPNC effects on patient activation, engagement, stress, and healthy behaviors and answer whether improving these maternal psychosocial and behavioral factors will explain the potential benefits of GPNC on birth outcomes or on racial disparities.

Our long-term goal is to improve prenatal care in the primary care setting to reduce the racial disparities in birth outcomes in a medically underserved population. Toward this long-term goal, **the objectives of this investigation are** 1) to compare birth outcomes as well as maternal behavioral and psychosocial outcomes by race among pregnant women who participate in GPNC, to women in the traditional individual prenatal care (IPNC) and 2) to investigate whether improving women's activation, engagement, and stress will explain the potential benefits of GPNC on birth outcomes and racial disparities.

C. Study Methodology

1. Study Design

This proposed study will employ **a prospective, randomized, controlled trial (RCT) design** conducted at a single center.

2. Comparison Groups: <u>There will be no administration of any therapeutic or prophylactic agent</u> in either group.

<u>1:</u> Group 1: CenteringPregnancy group prenatal care

- Pregnant women will receive prenatal care in a group medical setting.
- The CenteringPregnancy model (Centering Healthcare Institute, Cheshire, CT) has been implemented in over 100 clinical practices in the United States and abroad since 1995 and <u>is unlikely to cause major problems for pregnant women</u>.

<u>2:</u> Group 2: Individual prenatal care

- Pregnant women will receive prenatal care in a traditional, individual exam room setting.

3. Procedures:

<u>Identification of Potentially Eligible Patients</u>

Potentially eligible patients will be identified at the time of entry to prenatal care at the GHS OB-GYN Center.

FOR PATIENTS INITIATING CARE AT THE OB CENTER:

1. At the time of the nurse education visit at the GHS OB-GYN Center, all patients will be screened for study eligibility based on a series of screening questions answered by the GHS intake nurses. Most of these questions are already a routine part of the intake process for new patients coming in to the practice. Both eligibility and ineligibility will be documented for all new patients in the medical record.
    a. Women who are ineligible for the study will have research study status changed to "ineligible" in the research package in the Epic EMR, along with the reason for exclusion.
    b. All other women will have the research study status changed to "identified" in the research package in the Epic EMR.

2. At the time of the nurse education visit, patients will have baseline laboratory studies and ultrasound studies scheduled as a part of routine intake to prenatal care. These results will be reviewed by the research nurses, in order to verify study eligibility.

3. The final eligibility screening will take place when the patient returns to the OB Center for her second visit, which is scheduled with a healthcare provider. During this visit, either before or after the history and physical exam have been completed, the

research nurses will approach eligible patients for study enrollment. Patients will be taken to a quiet space to have the study explained.

FOR PATIENTS TRANSFERRING CARE IN TO THE OB CENTER, WHO INITIATED PRENATAL CARE WITH ANOTHER PRACTICE:

1. At the time of the nurse education visit at the GHS OB-GYN Center, all patients will be screened for study eligibility based on review of medical records and a series of screening questions answered by the GHS intake nurses. Most of these questions are already a routine part of the intake process for new patients coming in to the practice. Both eligibility and ineligibility will be documented for all new patients in the medical record.
    a. Women who are ineligible for the study will have research study status changed to “ineligible” in the research package in the Epic EMR, along with the reason for exclusion.
    b. All other women will have the research study status changed to “identified” in the research package in the Epic EMR.

2. The final eligibility screening will take place after the patient is scheduled with a healthcare provider. This may be the same day, or may be a separate day from the nurse education visit. Once the history and physical exam has been completed, the research nurses will approach eligible patients for study enrollment. Patients will be taken to a quiet space to have the study explained.

The study eligibility, recruitment, and follow-up procedure is summarized in Figure 1.

Figure 1. Study Flow Chart

Screening visit (Nurse Education visit)
(≤ 20 6/7 weeks' GA)

Education nurse (intake nurse) completes:

- Screening questions
- Medical record review
- Records "ineligible" (and reason) or "identified" status in EMR

→ **Exclude**

- <14 or >45 years
- > 20 6/7 weeks GA
- Medical, pregnancy, social/behavioral complications

↓

Baseline visit (First Provider visit or subsequent visit
≤ 23 6/7 weeks' GA)

Research nurse:

- Reviews lab and ultrasound results in EMR to verify eligibility for identified women
- Approaches eligible patients to explain study
- Completes written consent
- Conducts randomized allocation after asking stratification questions
- Has the participant complete survey 1and provides $25 incentive
- Provides group schedule to women randomized to CP or next appointment to IPNC
- Records ineligible/declined/enrolled status in EMR

→ **Exclude**

- <14 or >45 years
- > 23 6/7 weeks GA
- Medical, pregnancy, social/behavioral complications

↓

→ **Study Enrollment**

3rd Trimester Follow-up (at 32-36 weeks' appointment)

- Research nurse meets participants at the time of visit for survey 2 completion
- If participant has attended 5 visits in treatment arm, RN provides $50 incentive for study retention.

↓

Incentive follow-up

- Research nurse monitors participants for attendance at 5 or more visits in assigned treatment group, meets participant at visit to provide $50 incentive

↓

Postpartum Data Collection (at 12 weeks' postpartum)

- Research nurse (or other study staff) completes medical record review in RedCap

Study Enrollment

1. The patient is approached for study participation by research staff during a routine visit at the OB-GYN Center once the eligibility criteria have been met. This will typically be at the time of her first new patient visit with a healthcare provider, but may take place at any visit as long as she is within the eligible gestational age range.

2. The enrollment process involves a face-to-face interview with one of the study team for verification of study eligibility and counseling regarding study procedures, potential benefits and risks, prior to obtaining written consent.

Treatment Allocation

3. Research staff will complete a brief interview with patients to collect minimum demographic information in order to facilitate the randomization procedure (see Appendix A). This will include questions about race and ethnicity, and allow stratification into one of four groups; Hispanic, non-Hispanic Black, non-Hispanic White, and Other (which includes mixed-race).

4. Subjects allocated to “Group 1: CenteringPregnancy Group Prenatal Care” will be grouped (8-12 women who are all due to deliver in the same month) and will receive ten 2-hour group prenatal care sessions according to the standard curriculum provided by the Centering Healthcare Institute. Women will receive a patient workbook with health information and activities to promote goal setting and self-care, empowering them to become more engaged in their health care and managers of their health. During the first 30 minutes of each group session, a credentialed healthcare provider (nurse practitioner or nurse midwife) will conduct a brief physical assessment in a semi-private area of the group space; women measure and record their own weight and blood pressure. The healthcare provider will facilitate the group discussion for the remaining 90 minutes of each session. Topics discussed throughout sessions include pregnancy and nutrition, childbirth preparation, exercise, stress management, relationships, and parenting. Women randomized to GPNC will have access to additional IPNC visits (outside of the ten scheduled group sessions) as needed. The GPNC curriculum is design to be culturally appropriate and the study site can deliver the intervention in both English and Spanish. **The date of the first CenteringPregnancy session will be provided to the patient at the time of group assignment.**

5. Subjects allocated to “Group 2: Individual Prenatal Care” will receive standard, traditional individual prenatal care in accordance with the schedule of visits recommended by the American College of Obstetricians and Gynecologists.[40] Women will attend monthly provider visits for the first 28 weeks of pregnancy, every two to three weeks until 36 weeks, then weekly. Visits include an initial medical and psychosocial history, and ongoing physical assessment as well as patient education on pregnancy and prenatal care, options for intrapartum (delivery) care and educational programs, breastfeeding, and pediatrician selection. Women randomized to IPNC will not attend GPNC sessions. **Routine follow-up will be scheduled at the time of group assignment.**

Both groups: Women will receive routine screenings as well as specialized tests, interventions, and referrals depending on risk factors and the course of pregnancy.[40] Women who qualify for progesterone treatment will receive it; women with a history of PTB will also receive all relevant cervical length screenings.[41]

4. **Outcomes:**

   Specific Aim 1: To compare the rate of PTB (<37 weeks gestational age) and other selected birth outcomes (e.g. birth weight, caesarean section rate) among women who participate in GPNC to their counterparts in IPNC.

   Specific Aim 2: To compare the risk difference (RD) of PTB and other selected birth outcomes of Black women vs. White women in GPNC to the RDs of AA women vs. White women in IPNC.

   Specific Aim 3: To investigate the comparative effects of GPNC on patient activation, health behaviors, and stress.

5. **Study Population:** All medically low-risk women receiving prenatal care at the Greenville Health System OB/Gyn Center.

The target population consists of medically low-risk pregnant women. Women will be recruited at the GHS OB Center. The clinic population includes a large number of medically underserved women, with a 16.4% PTB rate and approximately 96% of deliveries covered by Medicaid. In our recent prospective study, the race distribution among 248 recruited women was 44.4% Blacks, 46.5% Whites, and 8.1% "Others". With regard to ethnicity, 87.9% was Non-Hispanic and 12.1% was Hispanic. We anticipate that our study population will have very similar demographic characteristics. Some differences of listed demographic characteristics between the GPNC and IPNC women are due to self-selection in the previous observational study. The proposed RCT design will assure that women assigned to the GPNC group have similar demographic distributions to women assigned to the IPNC group.

6. **Inclusion Criteria:**

   1) Patient age between 14-45 years
   2) Entry prenatal care before 20 6/7 weeks gestational age (defined as attendance at the intake screening visit). **Patients must be randomized by 23 6/7 weeks gestational age.**

7. **Exclusion Criteria (based on ACOG Committee Opinion #560, Medically Indicated Late-Preterm and Early Term Deliveries):**

   1) Medical complications of pregnancy that would preclude prenatal care provision by nurse practitioners or participation in group care
      - Pregestational diabetes,
      - Severe chronic hypertension requiring medication,
      - Morbid Obesity with BMI >45
      - Renal disease with baseline proteinuria >1g/24 hours
      - Any disease requiring chronic immunosuppression (SLE, solid organ transplant)
      - Active pulmonary tuberculosis
      - Sickle cell anemia
      - Human Immunodeficiency Virus Infection
      - Literacy level inadequate to complete the questionnaires in English or Spanish
      - Other medical conditions that would exclude women from group care at the discretion of the PI

   2) Pregnancy complications that would preclude prenatal care provision by nurse practitioners or participation in group care
      - Multiple gestation

- Lethal fetal anomalies
- Other pregnancy complications that would exclude women from group care at the discretion of the PI

3) Social and behavioral complications of pregnancy which would preclude prenatal care provision by nurse practitioners or participation in group care

- Current incarceration
- Severe psychiatric illness

8. Allocation Method

A computer generated randomization schedule with 1:1 allocation will be utilized with fixed block size of 4. These assignments will be stratified by race (Hispanic, Black, White, and Other and Mixed).

The research staff is responsible for accessing the centralized randomization system and obtaining the allocation assignment. Notification of randomization will be placed on patient chart with visit date for subsequent prenatal care visit for each treatment group.

9. Withdrawals, Losses and Deviations

A staff research nurse will be tasked with tracking follow-up in conjunction with the study investigators. Special efforts that will be taken to minimize the loss to follow-up include: 1) provide reminder calls (will be made 1 day before each study visit), 2) limit participant burden by having recruitment and survey activities conducted when women are already at the OB Center and provision of child care during group visits, 3) provision of an inviting research environment, 4) be on time and keep communication open, and 5) provide incentives/compensation ($50 if women complete 5 or more sessions/appointments in their assigned treatment condition).

Women who have a miscarriage or leave the practice and deliver their baby at a different hospital will be treated as lost to follow-up and will be handled appropriately in the primary analysis. However, we will make every effort to obtain data for women who deliver at other facilities within SC through obtaining their birth outcomes data through SC Department of Health and Environmental Control.

No matter the directions and the reasons of cross-over and regardless of the number of missed PNC visits, participants will be kept in their original assignments, as the primary analysis follows the intent-to-treatment approach.

10. Data Collection/Outcome Measurement

We plan to collect data at the following 3 visits: 1) Baseline Visit (typically scheduled within one week after the screening). At the time of Baseline Visit, women will undergo group assignment and complete the baseline data collection (Survey 1); 2) At 32-36 weeks, research nurses will meet participants prior to or after their appointment of prenatal care to administer the second survey (Survey 2); 3) Postpartum data collection will be conducted after women attend their 6-week postpartum visit and consist of medical chart abstraction, not requiring patient contact. The study surveys will be designed electronically by using RedCap. Patients' medical information will be collected by the Epic EMRt. Regular data review/reports will be generated through the Epic EMR to monitor the patient enrollment, group balance, and the time to make the reminding calls & patient incentives. The overall timeline for study visits and data collection is summarized in Table 1 below:

Table 1: Data Collection/Outcome Measurement	Gestational age <20 Survey 1	Gestational age 32-36 Survey 2	6-week PP Medical Chart Review
**STUDY OUTCOMES**			
**Birth outcomes and pregnancy complications**			
Preterm birth (gestational age, spontaneous or indicated)			X
Birth weight			X
APGAR scores			X
Admission to neonatal intensive care unit (NICU)			X
Intrauterine fetal demise			X
Neonatal death			X
Maternal anthropometric measures			X
Gestational weight gain			X
Pre-eclampsia			X
Gestational hypertension			X
Gestational diabetes			X
Intrauterine growth restriction			X
Macrosomia (weight >4000 g.)			X
Hospital admission during pregnancy, reason			X
Cesarean-section (primary or repeat)			X
Vaginal birth after Cesarean section			X
Induction and reason			X
**Maternal behavioral outcomes**			
Breastfeeding self-efficacy, intention, and knowledge		X	
Breastfeeding at hospital discharge and postpartum visit			X
Maternal smoking, alcohol and marijuana use	X	X	X
Stress management		X	
Physical Activity (IPAQ), dietary intake, and multivitamin use		X	
**Maternal psychosocial outcomes**			
Patient Activation Measure	X	X	
Readiness for labor and delivery		X	
Prenatal Planning and Preparation Coping		X	
Prenatal stress, anxiety, and depressive symptoms	X	X	
Support from baby's father		X	
**Interconception care**			
Attendance at postpartum visit			X
Postpartum contraception use			X
**COVARIATES**			
**Demographics**			
Race and ethnicity	X		
Age, income, education, marital status, insurance, employment, access to health care, household characteristics, address	X		
Use of other services in pregnancy			
**Women's life circumstances and stressors**			
Family support and relationship with baby's father	X		
Life stressors, including food insecurity, adverse childhood events, and life outlook and coping mechanisms	X		
Financial stress, housing instability, and food insecurity in pregnancy		X	
Everyday Discrimination Scale	X		
**Health and pregnancy history**			
Pre-pregnancy weight			X
Previous preterm birth			X
Reproductive tract infections in current pregnancy			X
Number of prior pregnancies, live births, and complications			X

PRENATAL CARE (PNC) PROCESS MEASURES			
Pregnancy-related empowerment, discrimination in prenatal care, group cohesion (for Centering participants), attendance of support people at prenatal care		X	

Data Accuracy and Protocol Compliance: Quality control will include regular data verification and protocol compliance checks by the PI's and will be completed on a monthly basis for the first 6 months and then on a quarterly basis afterward. Reports detailing the study progress and subject status, any adverse events, and any protocol deviations will be completed yearly. The PIs will be responsible for implementing and maintaining a quality management system with written development procedures to ensure that studies are conducted and data are generated, documented, and reported in compliance with the protocol. Protocol adherence will be monitored by both the GHS and the Clemson University IRB Committees.

All study staff members will be informed by the PI about any unanticipated problems involving risks to subjects or others. If any protocol changes are needed, the PI will submit a modification request to the IRB Protocol. Changes will not be implemented prior to IRB approval unless necessary to eliminate apparent immediate hazards to the research subjects. In such a case, the IRB will be promptly informed of the change following implementation (within 14 working days).

Statistical review of the study will be conducted by a statistician periodically during the intervention phase of the study. Interim analyses will be performed by the PI quarterly to assess outcomes. If an adverse event occurs, consideration will be given to stopping the study early. In the event of early conclusion of the study, the IRB will be promptly notified.

11. Study Size and Power

We propose to recruit and follow 3,160 (N=1,580 in each group) pregnant women.

We calculated our sample size based on Aims 1 & 2 with the primary outcome of PTB (<37 weeks). Based on the literature and our preliminary data, the rate of PTB was approximately 10-16% of women in the traditional care setting. The odds ratio of comparing PTB in GPNC to IPNC was 0.67 in the Ickovics study and 0.53 in our own study. Our own work also suggests that the risk difference (RD) of having PTB between Black women and White women was 1.0% in the IPNC and 2.4% in the GPNC (**Table 2**). Taken together, it is reasonable to assume that the relative risk of PTB in the IPNC group could range from 10% to 16%. **Table 3** gives the estimated minimum number (in each group) required to achieve an alpha of 0.05 and a power of 80% or 90% to detect the risk ratio from 0.5 to 0.7 in **Aim 1**, given that the proportion (P_0) of PTB rate in women at the IPNC group ranges from 10% to 16%. **Table 4** gives the estimated minimum number (in each group) of participants required achieving an alpha of 0.05 and a power of 80% or 90% to detect the RD from 0.01 to 0.08, given the rate difference (P_0) of preterm birth between Black women and White women at the IPNC group ranges from 2% to 3% in **Aim 2**. Therefore, for most reasonable assumptions of P_0, our study will need N = 2748 (1374 in each group) to detect reduction in risk difference of 1.4% between Black and White women in GPNC vs. IPNC group. With an estimated 15% attrition rate, we will target recruiting 3,160 (N=1580 in each group) women for this study. **This sample size estimates that the proposed study will have 90% power to detect racial difference on PTB for Aim 2.**

**Table 2.** Reference data from literature to calculate the sample size			
Reference	Parameters	IPNC	GPNC
Ickovics J, 2007	PTB rate	13.8%	9.8%
	OR or RR	0.67 (95% CI: 0.44-0.99)	
Picklesimer A, 2012	PTB rate	12.7%	7.9%
	OR or RR	0.53 (95% CI: 0.34-0.81)	
	RD	16.1% - 13.7% = 2.4%	7.5% - 6.5% = 1.0%

**Table 3.** Sample Size and Power Calculations Based on Aim 1						
	**Power = 80%**			**Power = 90%**		
	RR=0.50	RR=0.60	RR=0.70	RR=0.50	RR=0.60	RR=0.70
P_0=0.10	435	721	1356	582	965	1814
P_0=0.13	326	540	1014	436	723	1356
P_0=0.16	258	427	800	345	571	1070

**Table 4**. Sample Size and Power Calculations Based on Aim 2						
	**Power = 80%**			**Power = 90%**		
	RD=0.01	RD=0.014	RD=0.018	RD=0.01	RD=0.014	RD=0.018
P_0=0.02	2319	1027	526	3103	**1374**	704
P_0=0.03	3826	1799	995	5121	2048	1332
P_0: the RD of PTB in IPNC group; RR: relative risk						

Approximately 250 women begin prenatal care at the GHS OB Center each month, and 75% of them will be eligible for the proposed study (180 eligible women per month). Given our previous recruitment rate of 50%, the targeted study sample size will be achieved in 3 years. Recruitment is anticipated to begin October 2016 (or last quarter of the Year 1) and will be monitored monthly.

**Anticipated study recruitment**					
	Eligible # of women/month	Targeted recruitment rate	Targeted recruitment #/month	Targeted recruitment #/year	Targeted recruitment # for 3 years
GPNC Recruitment	90	50%	45	540	1620
IPNC Recruitment	90	50%	45	540	1620

12. Analytic Plan

Statistical analyses plan
Primary analyses for Aims 1, 2, and 3 will be conducted using an intent-to-treat approach. Study participants will be retained in their original assignment groups after the randomization in the analysis regardless of the number of missed visits, the use of additional services (e.g., progesterone), or lost to follow-up.

Preliminary analysis: Preliminary analysis will involve 1) generation of new variables and assessing missing values and 2) descriptive statistics. Descriptive analyses will be conducted for all variables and the amount of missing data will be assessed. Achievement of randomization will be evaluated through the comparison of baseline key variables between GPNC and IPNC groups. Baseline key characteristics will also be compared between eligible women who participate the study and who don't participate to examine the potential for bias.

Aim 1 analysis plan: comparison of primary and secondary outcomes for GPNC vs. IPNC
To test the hypothesis for aim 1 that women who participated in GPNC will have a lower rate of PTB and other selected birth outcomes as compared to their counterparts in IPNC, the

multivariate regression models (logistic regression if the outcome is a binary variable and linear regression if the outcome is a continuous variable) will be employed with the intervention assignment as the primary independent variable. Stratified analyses will be conducted (e.g. race, previous history of PTB) to compare the primary and other outcomes within sub-groups.

Using the primary outcome of PTB as an example, the regression equation will be:

(1) $\text{logit}(Y) = \beta'_0 + \beta'_1 G$

Here, Y is the PTB (Y=1 if delivery <37 weeks; Y=0 if delivery ≥37 weeks). G is the indicator variable for the group assignment (G=0 if in IPNC; G=1 if in the GPNC). $\beta_{0,}$ and β_1 are regression coefficients.

Therefore, $e^{\beta'0}$ represents the odds of having PTB for women in IPNC (equation 1). $e^{\beta'0 + \beta'1}$ represents the odds of having PTB for women in GPNC. $e^{\beta'1}$ represents the odds ratio of having PTB for women in the GPNC group compared with women in the IPNC group (control group), therefore, represents the estimate of the overall intervention effect for both white and AA women. <u>We will test the null hypothesis: $\beta'_1 = 0$.</u>

Aim 2 analysis plan: comparison of racial disparities of primary and secondary outcomes for GPNC vs. IPNC

To test the hypothesis for Aim 2 that the Black-White differences in PTB and other selected birth outcomes is smaller in the GPNC as compared with that in IPNC, we will apply analyses similar to Aim 1 but using the following multivariate regression models (logistic regression if the outcome is a binary variable and linear regression if the outcome is a continuous variable). Using the primary outcome of PTB as an example, the regression equation will be:

(2) $\text{logit}(Y) = \beta_0 + \beta_1 G + \beta_2 R + \beta_3 G^*R$

Here again, Y is PTB (Y=1 if delivery <37 weeks; Y=0 if delivery ≥37 weeks) and G is the indicator variable for the group assignment (G=0 if in IPNC; G=1 if in the GPNC). In addition to equation (1), we add two more explanatory variables in the equation (2): R is the indicator variable for the race (R=0 if race is White; G=1 if race is Black); G*R is the interaction term for G and R. If we separate women by intervention and race groups, we will have the following questions:

<u>For White women in the IPNC group</u>: (2.1) $\text{logit}(Y) = \beta_0$

<u>For White women in the GPNC group</u>: (2.2) $\text{logit}(Y) = \beta_0 + \beta_1$

<u>For Black women in the IPNC group</u>: (2.3) $\text{logit}(Y) = \beta_0 + \beta_2$

<u>For Black women in the GPNC group</u>: (2.4) $\text{logit}(Y) = \beta_0 + \beta_1 + \beta_2 + \beta_3$

Therefore, $e^{\beta 0}$ represents odds of having PTB for White women in IPNC group. $e^{\beta 0 + \beta 1}$ represents odds of having PTB for White women in GPNC group. $e^{\beta 1}$ represents the odds ratio of having PTB for White women in the GPNC group compared with White women in the IPNC group (control group), therefore, represents the estimate of the intervention effect for White women. $e^{\beta 0 + \beta 2}$ represents odds of having PTB for Black women in the IPNC group. $e^{\beta 0 + \beta 1 + \beta 2 + \beta 3}$ represents the odds of having PTB for Black women in the GPNC group. $e^{\beta 1 + \beta 3}$ represents the odds ratio of having PTB for Black women in the GPNC group compared to Black women in the IPNC group (control group), therefore, representing the estimate of the intervention effect for Black women. $e^{\beta 2}$ is the odds ratio of having PTB for Black women compared with White women in the IPNC group. $e^{\beta 2 + \beta 3}$ is the odds ratio of having PTB for Black women compared with White women in GPNC group. Therefore, $e^{\beta 3}$ is odds ratio of having PTB for Black vs. White women in GPNC group compare with that in the GPNC group.

<u>We will test the null hypothesis: $\beta_3 = 0$.</u>

For aim 2, only women identified themselves as either Black or White will be included. Therefore, women with self-identified race as “Others” will be excluded in this analysis. We estimate that 8-10% will be in the “Other” category of race.

Aim 3 analysis plan

Aim 3 is to compare the effects of GPNC on maternal psychosocial (i.e. activation, engagement, stress) and behavioral (i.e. smoking, healthy eating, healthy practices) changes

and to explore whether improving maternal psychosocial and behavioral outcomes will explain the potential benefits of the GPNC on racial disparities in birth outcomes. To address Aim 3, we will first to test whether changes in maternal behavioral or psychosocial factors differed by intervention and race. To do this, a similar analytic plan as used in Aim 1 & 2 will be applied, however, the dependent variables will be changes of behavioral or psychosocial factors from baseline (~ 20 weeks) visit to late pregnancy (30-32 weeks). If we observe any significant differences in these behavioral or psychosocial factors by intervention or race, the next step is to explore whether and to what extent the differences in the intermediate behavioral or psychosocial factors could explain the intervention effect or racial disparities of birth outcomes. To do so, the main multivariate regression models will be examined with and without adjustment for the intermediate behavioral or psychosocial variables.

Additional analyses

In addition to the primary intent-to-treatment analysis and regression models, several alternative approaches will also be conducted to gain an in-depth evaluation of the GPNC model.

- **Sensitivity analyses:** Multiple sensitivity analyses will be conducted. We will compare the intervention effect within women who receive an adequate number of prenatal care visits defined by the Kotelchuck index. We will also evaluate whether there are dose-response intervention effects by examine the association between the primary outcome variables and the number of visits during prenatal care. Since women who self-identify with Hispanic ethnicity have a similar PTB rate to White women from the literature and in our practice, the Hispanic women will not be analyzed separately in the primary analysis (without necessarily adding additional insights into the racial disparities in rates of PTB). However, sensitivity analysis will be conducted according to the Hispanic ethnicity.

- **Cox proportional hazard model**: Cox proportional hazard models will be performed to assess time to preterm delivery between the GPNC and IPNC groups.

- **Percentage of excess risk of PTB in Black women that can be reduced by GPNC**: If the results in Aim 2 are significant, indicating that GPNC reduces the racial disparity of PTB between Black and White, we will further quantify the extent to which GPNC would explain Black-White differences in PTB. We will calculate the percentage of excess risk of PTB explained by GPNC using the following formula: % excess risk=(RR_1-RR_2)/(RR_1-1) where RR_1 is the relative risk (RR) of PTB for Blacks vs. Whites in the main model without adjustment for GPNC intervention and RR_2 is the RR in the model with adjustment for GPNC.[67, 68] The % excess risk reflects the percentage of racial difference in PTB between Blacks and Whites that can be reduced by GPNC intervention.

D. Possible Risks

Minimal risk to the patient is associated with this study, since patients in both arms will receive prenatal care which meets or exceeds the standards set by the American College of Obstetricians and Gynecologists. The most likely risk to the patient would be loss of confidentially.

E. Possible Benefits

Based on available clinical data there is good probability that the participants in the group prenatal care arm of the study (Group 1) will benefit directly from the study by demonstrating few preterm deliveries and having fewer infants that require admission to the neonatal intensive care unit. The study is also expected to yield generalizable knowledge which contributes to the field of obstetrics and gynecology.

F. Special Precautions

Patients will be given adequate time to review the consent form after being counseled on the study, and will be given adequate time to have questions and concerns answered.

Research staff will be trained in protection of patient confidentially. Captured data will be maintained on password protected electronic files, accessed through password protected computers that are housed behind locked office doors.

Mechanism for Reporting Adverse Event: We plan to report any adverse event to the IRB and the NIH as appropriate. An adverse event report form will be generated and used for collecting adverse events during the study period. Throughout the study, the PIs (One PI is the Director and leading physician in the study site) will monitor the participants for adverse events. Events determined by the PIs to be unanticipated problems involving risks to subjects or others will be reported the IRB as in accordance with IRB policy. Adverse events that are determined by the PI to not be unanticipated problems involving risks to subjects or others will be reported per IRB policy at the time of continuing review. In addition, NIH will be notified of by the PIs of all adverse events.

G. Procedures to Maintain Confidentiality

Individual patient data collected as part of this investigation will remain confidential. Composite results from this investigation, however, will be disseminated to the scientific community. The data sheet will contain the patient’s name and medical record number for later chart review. Actual data collection will only be performed by the investigators for their study or their designated research personnel. Once the data is collected, each patient will be assigned an alpha-numeric identifier which will allow entry of de-identified data in a computer database. The original data sheets will be stored in a secure location in the Department of Obstetrics and Gynecology.

APPENDIX A

Treatment Allocation Interview Questions

What is your race or origin (you can answer one or more)?

- White
- Black or African American
- Hispanic, Latino, or Spanish origin (for example, Mexican, Mexican American, Puerto Rican)
- Some other race or origin (write in response)

Are you of Hispanic, Latino or Spanish origin?

◊ No, not of Hispanic, Latino or Spanish origin

◊ Yes, Mexican, Mexican American, Chicano
◊ Yes, Puerto Rican
◊ Yes, Cuban
◊ Yes, another Hispanic Latino or Spanish origin – Point of origin, for example, Argentinean, Colombian, Dominican, Nicaraguan, Salvadoran, and so on.

What is your race? (may choose one or more)

◊ White
◊ Black, African American or Negro
◊ American Indian or Alaskan Native
◊ Pacific Islander
◊ Asian American
◊ Some other race

Statistical Analysis Plan for Primary Outcome Analysis of CRADLE project

Drafted on August 31, 2020

By

The CRADLE Research Group

Table of Contents

1 Introduction 3
2 Components of the Two Study Arms 4
2.1 GPNC (intervention) Arm 4
2.2 IPNC (control) Arm 4
2.3 Both Arms 4
3 Definitions of Primary Outcome Measures 4
3.1 Preterm Birth (PTB): 4
3.2 Low Birth Weight (LBW): 5
4 Definitions of Study Analytic Sample 5
4.1 Primary *modified* intention-to-treat (mITT) analytic sample: 5
4.2 Intention-to-treat analytic sample: 6
4.3 Per-compliance analytic sample: 6
4.4 As-treated analytic sample: 6
4.5 Significance level and software 6
5 Predictors, Baseline characteristics, and Covariates 7
5.1 Predictors 7
5.2 Baseline Characteristics 7
5.3 Comparisons 9
5.4 Adjusting covariates 9
6 Analytic plans for the primary outcomes 9
6.1 Primary Study hypothesis 9
6.2 Statistical models 9
6.3 Effectiveness measures 10
6.4 Handling missing data 10
6.5 Statistical Power 10
6.6 Sensitivity analysis 11
7 Subgroup analysis 11
7.1 Subgroups to be compared: 11
7.2 Statistical Models 12
8 Appendix 13

1 Introduction

The present statistical analysis plan is prepared for the Cradle study main outcome manuscript. The Cradle study is a large, single site randomized clinical trial (RCT) that aims to test the effectiveness of CenteringPregnancy Group Prenatal Care (GPNC) in comparison with the current standard model of Individual Prenatal Care (IPNC). The Cradle study is sponsored by the *Eunice Kennedy Shriver* National Institute of Child Health and Human Development (R01HD082311; Clinical Trial Registration #: NCT02640638). The primary outcomes are preterm birth (PTB, delivery <37 weeks of gestational age) and low birthweight (LBW, birth weight <2500 grams). The primary aim of the Cradle study is to test hypotheses that compared to the participants randomized to the IPNC arm those in the GPNC arm will: 1) improve PTB and LBW; and 2) reduce the racial disparity in those two outcomes between Blacks and Whites.

To test these hypotheses, more than 3000 mother-baby dyads were planned to be recruited and randomly assigned to GPNC or IPNC with a 1:1 ratio stratified by self-identified race. Eligible pregnant women will be enrolled before 20 6/7 gestational weeks and followed until delivery and post-partum. The study site is the Prisma Health Greenville Memorial Hospital OB/Gyn Center prenatal primary care clinic in Greenville, South Carolina. The intentions of this statistical analysis plan are to guide data analysts to comply with for maximizing transparency and reproducibility of the statistical analysis results and minimizing potential biases by not being influenced by results. This plan is prepared and approved by the entire Cradle study investigator team prior to the completion of main outcome data collection and cleaning which is anticipated to be accomplished by December 2020.

This SAP addresses Aims 1 and 2. Aim 3 will be addressed through separate papers approved by the Cradle Publications and Presentations Committee (PPC). The specific aims as detailed in the proposal are:

Aim 1: To compare the rate of PTB (<37 weeks gestational age) and other selected birth outcomes and pregnancy complications (e.g. birth weight, caesarean section rate) among women who participate in GPNC to their counterparts in IPNC.

Aim 2: To compare the risk differences (RDs) of PTB and other selected birth outcomes and pregnancy complications of Black women vs. White women in GPNC to the RDs of Black women vs. White women in IPNC.

Aim 3: To compare whether women in GPNC have improved maternal psychosocial (i.e. activation, engagement, stress) and behavioral (i.e. smoking, healthy eating, health practices) outcomes as compared to their counterparts in IPNC and to explore whether improving certain maternal psychosocial and behavioral outcomes will explain the potential benefits of GPNC on racial disparities in birth outcomes.

.

2 Components of the Two Study Arms

2.1 GPNC (intervention) Arm

Women will be grouped (8-12 women who are all due to deliver in the same month) and will receive ***ten*** 2-hour group prenatal care sessions according to the standard curriculum provided by the Centering Healthcare Institute. Women will receive a patient workbook with health information and activities to promote goal setting and self-care, empowering them to become more engaged in their health care and managers of their health. During the first 30 minutes of each group session, a credentialed healthcare provider (nurse practitioner or nurse midwife) will conduct a brief physical assessment in a semi-private area of the group space; women measure and record their own weight and blood pressure. The healthcare provider will facilitate the group discussion for the remaining 90 minutes of each session. Topics discussed throughout sessions include pregnancy and nutrition, childbirth preparation, exercise, stress management, relationships, and parenting. Women randomized to GPNC will have access to additional IPNC visits (outside of the ten scheduled group sessions) as needed. The GPNC curriculum is designed to be culturally appropriate and the study site can deliver the intervention in both English and Spanish.

2.2 IPNC (control) Arm

Women will receive standard, traditional individual prenatal care in accordance with the schedule of visits recommended by the American College of Obstetricians and Gynecologists. Women will attend *monthly provider visits* for the first 28 weeks of pregnancy, every two to three weeks until 36 weeks, then weekly. Visits include an initial medical and psychosocial history, and ongoing physical assessment as well as patient education on pregnancy and prenatal care, options for intrapartum (delivery) care and educational programs, breastfeeding, and pediatrician selection. Women randomized to IPNC will not attend GPNC sessions.

2.3 Both Arms

Women will receive routine screenings as well as specialized tests, interventions, and referrals depending on risk factors and the course of pregnancy. Women who qualify for progesterone treatment will receive it; women with a history of PTB will also receive all relevant cervical length screenings.

3 Definitions of Primary Outcome Measures

3.1 Preterm Birth (PTB):

All pregnancies have pregnancy dating confirmed by ultrasound performed <20 weeks gestational age, which is used to establish the estimated date of delivery (ACOG Committee Opinion #700, 2017). This is considered the best obstetrical estimate of gestational age. The gestational age at delivery is calculated based on date of delivery abstracted from the medical record and the best obstetrical estimate of the estimated date of delivery. Preterm birth is defined as any delivery <37 weeks of gestational age, according to the Centers for Disease Control and the National Center for Health Statistics definition. This

definition of PTB in our study will include both indicated and spontaneous PTB. Miscarriage will be declared if women deliver at <20 weeks gestational age and will not be considered preterm birth and thus be treated as missing with respect to PTB. That threshold will serve as the primary criterion for miscarriage. Secondarily, however, we also plan to consider pregnancies ending 20 – 22 weeks gestational age as miscarriages for a sensitivity analysis since infants delivering <23 weeks gestational age are not offered resuscitation in our institution, Prisma Health, and the literature varies about treating these pregnancies as a preterm birth vs. miscarriage   Although the binary PTB outcome will serve as the primary outcome definition, time to birth on a survival outcome scale will also be analyzed; for this analysis missing gestational age will be treated as right censored at the gestational age at the last time point of visit.

3.2 Low Birth Weight (LBW):

Low birthweight (LBW) is defined as any infant weighing <2500 grams using the first weight recorded at the time of birth, following the definitions from the Centers for Disease Control and the National Centers for Health Statistics. This LBW group is further sub-divided into extremely low birthweight (ELBW <1000 grams), very low birthweight (VLBW, <1500 grams), moderately low birthweight (MLBW 1500 – 2499). The weight at birth will be extracted from the Prisma Health site electronic health record system and manually reviewed by the research team to validate the low birth weight birth outcome. Again, although the binary LBW outcome will serve as the primary outcome definition, actual birth weight will be compared between GPNC and IPNC.

4 Definitions of Study Analytic Sample

All study participants must meet the screening inclusion and exclusion criteria AND sign the consent form. These participants are defined as enrolled participants/sample. From these enrolled participants, the analytic sample for all statistical analysis will be defined in the following categories.

4.1 Primary *modified* intention-to-treat (mITT) analytic sample:

This primary analytic sample includes the enrolled study participants who were: 1) randomized; 2) initiated interventions in the assigned study arm (GPNC or IPNC); and 3) birth outcomes are available and validated by chart review. This sample is referred herein to as the *modified* intention-to-treat (mITT) analytic sample. As per the first condition, the intention-to-treat principle reflects the principle that once the intervention was randomly assigned to a participant, the participant’s intervention assignment will not change in the analysis even if mothers crossed over to the other intervention during the trial period or did not complete intervention activities; that is, a binary indicator for the randomized (as opposed to received) care will be the predictor. As per the second condition, the participants who initiated the intervention will consist of those who we will be able to ascertain have attended at least one session of prenatal care in the assigned study arm after randomization. As per the third condition, we anticipate that delivery outcomes from patients who are transferred to other clinics may not be available. Since the majority of local hospitals are linked within our electronic medical record system, we have obtained permission to request medical records for women delivering outside of our health system, and will do our best to obtain these whenever possible. If patients birth outcome data including gestational age and

weight at delivery are not available, we will categorize such patients as lost to follow-up and will exclude them from the mITT sample.

4.2 Intention-to-treat analytic sample:

The intention-to-treat analysis sample included all randomized participants, and the binary indictor for the randomized care will be the predictor. This sample will serve for sensitivity analyses. To this end, missing outcome may need to be imputed for a sensitivity analysis (see below section 6.4 for handling missing outcome). However, analysis of survival time to birth would not need any imputation.

4.3 Per-compliance analytic sample:

This per-compliance analytic sample includes the enrolled study participants who 1) were randomized; 2) initiated treatment in the assigned study arm; and 3) birth outcomes are available and validated; 4) complied with the randomly assigned models of care, where no women assigned to IPNC received any GPNC sessions or vice versa and 5) attended at least 5 care sessions in the assigned study arm (GPNC or IPNC). Because GPNC participants will have some exposure to IPNC (e.g. at the beginning of pregnancy before GPNC starts, to supplement a missed GPNC session, and at end of pregnancy after session 10 but before birth), crossover from GPNC to IPNC is essential practice and will occur for all women randomly assigned to GPNC. This type of necessary crossover will not be considered a violation of compliance. This sample is referred to as the per-compliance (PC) analytic sample. The PC sample will be a subset of the mITT sample, and thus the indicator for the randomized care will be the predictor.

4.4 As-treated analytic sample:

We anticipate that there will be cross over to the other type of care than the randomized care. In such a case, we will use the received care as the predictor in this sample, and refer to as the as-treated (AT) analytic sample. When a patient had been exposed to both cares, we will take the more exposed care as received care; when they are equal, we will take the later care. However, the necessary IPNC care for the GPNC patients described in the above section 4.3 will not be considered crossover. AT sample will be determined based on the above ITT.

If nonadherence to study protocol is substantial, we will classify patients according to the amount of intervention received, with the number of group visits (range=0–10) as the primary predictor. We will identify characteristics associated with group attendance and include them in adjusted models for testing associations between the number of group visits and birth outcomes.

4.5 Significance level and software

All statistical analyses proposed in the present SAP will be repeated for each analytic sample. Comparison across all above analytic samples will be compared to assess internal or external validities or limitations of the study findings. All statistical analyses will be conducted using SAS v9.4 or higher. A two-tailed significance level of 0.05 will be applied to all statistical hypothesis testing and estimations of confidence intervals.

5 Predictors, Baseline characteristics, and Covariates

5.1 Predictors

The study arm: The study arm will be the predictor. However, its constitution will depend on the types of the analysis samples as described in the preceding section. In short, the predictor will be the indicator for the randomized or received care depending on the type of the analytic samples.

Race: At the time of randomization, on the initial demographics survey, women were asked by study personnel to self-identify race/ethnicity by choosing one of the following categories (“race”); Black/African American, White, Latina, Mixed and Other. These categories were used to stratify the study sample during randomization. Subsequently, using the same survey instrument, women were asked by study personnel to describe their ethnicity (Hispanic or non-Hispanic, “ethnicitynew”) and then their race (Black/African American, White, American Indian/Alaska Native, Asian, Native Hawaiian/Pacific Islander, Mixed or Other, “racenew”) in two separate questions following the definitions used by the US Census. Unfortunately, this question was inadvertently excluded from the initial survey instrument and this result is not available for the first 758 women enrolled in the project. Finally, in survey one, women were allowed to self-identify race and ethnicity in a series of questions. The first is a combined question including race and ethnicity, but that allows women to select more than one category (White, Hispanic, Black, American Indian, Asian, Middle Eastern, Pacific Islander, other or prefer not to answer, “racethn”).

For the disparity analyses, we will combine race/ethnicity into the following four categories considering Hispanic a race group rather than an ethnicity group: Whites, Blacks, Hispanics and Other. To this end, we will first use the survey 1 combined race and ethnicity categorization (“racethn”, where women could select more than one race/ethnicity designation) and if the response is missing then we replace by the race self-reported at randomization. In addition, comparison between Hispanic and non-Hispanic ethnic background will also be made. Specifically:

- For women who identified as Black or African American, assign as Black (i.e., even if they selected another designation below women who select Black or African American are assigned to this category)
- For women who identified as Hispanic, Latina or Spanish origin, assign as Hispanic (i.e, even if they selected another designation in addition to Hispanic, they will be assigned to this category).
- For women who identified as White, assign as White.
- For women who prefer not to identify, assign according to the response to “race” questionnaire item at randomization
- All other categories are coded as "other"

5.2 Baseline Characteristics

We will consider the following baseline characteristics to characterize the Cradle study participants

Demographic Characteristics:

Maternal race/ethnicity: Black; Hispanic; White; Other

Survey language: Spanish or English

Income, adjusted for household size (Using US Census Bureau Equivalence Scale; https://www.census.gov/topics/income-poverty/income-inequality/about/metrics/equivalence.html Note that approx. 22% responded don't know/not sure and another 8% prefer not to answer): Ordinal

Maternal age at study enrollment: ≤17; 18-25; 26-34; ≥35

Married; Married/engaged vs other

Education level achieved: HS- vs HS+

Employment status: Employed (Working full time or part time); Unemployed (laid off, looking for work); Keeping house (caring for family full time + family/disability leave)

Medicaid enrolled (payor 1 at time of delivery): Private, public, other (including none)

Lack of health insurance in the previous 12 months: Yes/no

Behvaioral charateristics

Cigarette smoking in pregnancy (How many cigarettes do you smoke on an average day now?):

Yes/no in lieu of Current smoking

Alcohol use in pregnancy Yes/no

Pregnancy intention: Yes/no

Clinical characteristics

Parity: Nulliparous vs. ≥1 other child

History of previous preterm birth: Yes or no from record review)

History of previous prehypertension

Infections in pregnancy (chlamydia, gonorrhea, trichomonas, bacterial vaginosis): Yes/no for each infection; and any vs. none

Any vaginal bleeding in pregnancy; Yes/no

History of prior cervical surgery (LEEP, cone biopsy) Yes/no

Cervical shortening (≤25 mm) identified on ultrasound 16-28 weeks: Yes/no

Cerclage placed during pregnancy: Yes/no

Uterine anomaly: Yes/no

Pregnancy conceived with assistive reproductive technology: Yes/no

Prepregnancy BMI: Undeweight (<18.5); Normal (18.5-<25); Overweight (25-<30); Obese (30+);

Entry to prenatal care, weeks (mean ± SD)

Gestational age at study entry, weeks (mean ± SD)

These definitions and classifications are displayed in Table 1 shell in the appendix. Although additional survey and chart review variables are detailed in a separate data dictionary, survey data used in this SAP is from survey 1 only. The data collected at enrollment using patient survey instruments will serve as the primary data sources for the baseline characteristics. The contents of Table 1 will be computed based on both the ITT and mITT samples.

5.3 Comparisons

First, the distributions of all baseline characteristic variables will be examined using graphical or descriptive statistics to identify any values out of range. When identified, out of range values will be found in the original record, compared and corrected if needed. Second, the success of randomization will be verified by comparing baseline characteristics between the two models of care arms, GPNC and IPNC among the ITT sample; the comparison will also be made in the mITT sample. Continuous variables will be compared between arms using t-tests or Mann-Whitney tests, and categorical variables will be compared using chi-square or Fisher's exact tests.

5.4 Adjusting covariates

The CONSORT guideline recommends that 1) only descriptive statistics on the baseline characteristics of *randomized* trials between arms without reporting of p-values following be described in a table, and 2) any baseline characteristics other than randomization stratification variables should not be included in comparing the randomized arms. Nevertheless, we will provide p-values for the mITT sample since this sample may no longer be a randomized sample. Furthermore, the imbalance of baseline characteristics among the mITT sample are anticipated and will be declared so if their p-values are less than a stringent level of 0.01, and such characteristics will be included in the statistical models testing the primary study hypotheses for adjusting purposes.

6 Analytic plans for the primary outcomes

6.1 Primary Study hypothesis

Effectiveness Hypothesis: *Women who participate in GPNC will have a lower rate of PTB and LBW as compared to their counterparts in IPNC.*

Disparity Hypothesis: *Differences in PTB and LBW between Black, White and Hispanic women among the GPNC participants is smaller than those in the IPNC participants.*

6.2 Statistical models

Effectiveness Hypothesis Testing: Multivariable logistic regression will be applied to test the overall effectiveness of GPNC vs. IPNC on the two the primary outcomes: PTB (<37 vs. >=37 weeks) and LBW (<2500 vs. >= 2500 grams). The arm indicator will serve as the primary predictor, and any unbalanced baseline characteristics will be included as covariates for adjusting purposes in the following multivariable logistic regression model.

(1) Logit(P(PTB or LBW)) = b0 + b1*GPNC + b2*Covariates

The testing effectiveness hypothesis will be equivalent to testing significance of the regression coefficient b1.

Disparity Hypothesis Testing: Multivariable logistic regression will again be applied to test the disparity effectiveness of GPNC vs. IPNC across Blacks, Whites and Hispanic (collectively "Race") on the two the primary outcomes: PTB (<37 vs. >=37 weeks) and LBW (<2500vs. >= 2500 grams). The interaction between the arm indicator and race (which will consist of two dummy variables representing Blacks and Hispanics while Whites will be serving as the referent group) will be tested in the following multivariable model, and again any unbalanced baseline characteristics will be included as covariates for adjusting purpose.

(2) Logit(P(PTB or LBW)) = b0 + b1*GPNC + b2*Race + b3*GPNC*Race + b4*Covariates

In short, the testing disparity hypothesis will be equivalent to testing significance of the interaction regression coefficient b3.

6.3 Effectiveness measures

For the results from testing the effectiveness hypothesis, we will report the odds-ratio (OR) of GPNC on the outcomes for each race group along with its p-value and 95% confidence interval. For the results from testing the disparity hypothesis, we will report a p-value based on df=2 Wald chi-square test for testing significance of the coefficient b3.

6.4 Handling missing data

Study participants might have a miscarriage or leave the study practice and deliver their baby at a different hospital. Although we will make every effort to obtain data for women who deliver at other facilities, we anticipate missing primary outcomes from such participants. This necessitates imputation for missing outcomes for the analysis of intention-to-treat analytic sample, if not for the other types of samples. To this end, we will apply a chained multiple imputation method separately for each arm; the imputation model would include baseline characteristics (specifics to be determined) as predictors. For example, we will impute five times for the missing data on the continuous scales of both gestational week and weight at birth, then generate the imputed binary outcomes based on those imputed continuous outcomes. Analysis of survival time to birth would not need any imputation.

6.5 Statistical Power

Based on the literature and our preliminary data, the rate of PTB was approximately 10-16% of US women in the traditional care setting. The odds ratio of comparing PTB in GPNC to IPNC was 0.67 in a published study and 0.53 in our own prior study. Therefore, it is reasonable to assume that the relative risk (RR) of PTB in the IPNC group could range from 10% to 16%. The estimated minimum number/sample size per arm of participants required to achieve an alpha of 0.05 and a power of 80% or 90% to detect the risk ratio (RR) from 0.5 to 0.7 ranged 435 to 1070 (Table 2) under the assumption that the proportion of PTB rate (P_0) in women at the IPNC group ranges from 10% to 16%. Our own work also

suggests that the risk difference (RD) of having PTB between Black women and White women was 1.0% in the IPNC and 2.4% in the GPNC cohort.

Assuming that the rate difference (P_0) of preterm birth between Black women and White women at the IPNC group ranges from 2% to 3%, the estimated minimum number/sample size (in each group) of participants required achieving an alpha of 0.05 and a power of 80% or 90% to detect the RD from 0.01 to 0.018 ranged 526 to 5121 (Table 3). Therefore, for most reasonable assumptions of P0, our study will need N = 2748 (1374 in each group) to detect reduction in risk difference of 1.4% between Black and White women in GPNC vs. IPNC group. With an estimated 15% attrition rate, we will target recruiting 3,160 (N=1580 in each group) women for this study.

**Table 2.** Sample Size and Power Calculations Based on Aim 1						
	**Power = 80%**			**Power = 90%**		
	RR=0.50	RR=0.60	RR=0.70	RR=0.50	RR=0.60	RR=0.70
P0=0.10	435	721	1356	582	965	1814
P0=0.13	326	540	1014	436	723	1356
P0=0.16	258	427	800	345	571	1070
P_0: PTB rate (P_0) in women at the IPNC group. RR: Risk Ratio						

**Table 3**. Sample Size and Power Calculations for testing Disparity hypothesis						
	**Power = 80%**			**Power = 90%**		
	RD=0.01	RD=0.014	RD=0.018	RD=0.01	RD=0.014	RD=0.018
P0=0.02	2319	1027	526	3103	**1374**	704
P0=0.03	3826	1799	995	5121	2048	1332
P0: RD of PTB between Blacks and Whites in IPNC arm. RD: Risk Difference						

6.6 Sensitivity analysis

Time to birth/delivery data: Cox proportional hazard models will be performed to assess time to birth/delivery on survival time scale of gestational week (as opposed to binary PTB) between the GPNC and IPNC groups for testing both hypotheses using the ITT sample without imputations adjusting for the covariates as defined above.

Continuous outcome data: For the analysis of continuous gestational age at birth and infant weight at birth, we will apply quantile or linear regression model for testing both hypotheses using all types of analytic samples adjusting for the covariates.

7 Subgroup analysis

Subgroup analysis will be conducted to identify subgroups in which the primary outcomes, PTB and LWB, are significantly different between GPNC and IPNC. In addition, testing equality of GPNC effects across subgroups will be conducted. This subgroups analysis will be conducted for both mITT and ITT samples.

7.1 Subgroups to be compared:

All subgroups defined by each level of all baseline demographic, clinical, and behavioral characteristics described in 5.2 will be examined. For instance, we will estimate test significance of effect of GPNC (vs. IPNC) on outcomes among employed participants and also among unemployed participants.

To test equality of the intervention effects between levels of subgroups, we will test interaction between the care and the levels of a subgroup.

7.2 Statistical Models

Estimates of treatment effects in terms of adjusted ORs and 95% CIs will be obtained separately in all subgroups using logistic regression models adjusting for the covariates. We will not include unbalanced covariates in multivariable models in these subgroups analysis if they serve as a subgroup. In short, for all subgroup analysis, we will apply a consistent modeling framework adjusting only for the study arm and the covariates that don't serve as subgroups.

Main intervention effect in subgroups: The main effect of GPNC effect will be assessed in a subgroup (eg, unemployed mothers)

(3) Logit(P(PTB or LBW)) = b0 + b1* GPNC + b2*Covariates.

The primary interest of this subgroup analysis will be estimating the GPNC vs. IPNC effect on the PTB and LWB outcome in terms of point (i.e., odds-ratio) and interval estimates (i.e., 95%CI) and testing significance of GPNC vs. IPNC effects in each group. The analysis results across all subgroups will be graphically summarized displayed in a forest plot (Figure 1).

Assessment of equality/heterogeneity of models care effect across subgroup levels: The following multivariable model will be used to test equality of GPNC effect between subgroups defined by baseline characteristics.

Logit(P(PTB or LBW)) = b0 + b1*Subgroup indicator+ b2*GPNC + b3*Subgroup indicator*GPNC + b4*Covariates

For examples, the value of "subgroup indicator" below will be 1 for employed and 0 for unemployed. The testing of equality/heterogeneity will be accomplished by be testing significance of the interaction term b3 that represents a difference in GPNC vs. IPNC effect on PTB or LBW between subgroups. Wald chi-square test will be used for testing significance of the coefficient b3.

8 Appendix

Table 1. Baseline characteristics of the Cradle study participants. (To be prepared for both ITT and mITT samples)

	CenteringPregnancy	Individual care	p
	xxx (xx.x%)	xxx (xx.x%)	
**Sociodemographic Characteristics**			
**Race**, %			
Black			
White			
Hispanic			
Other			
**Maternal age**, (mean ± SD)			
**Language**, English %			
**Education**, High school or above %			
**Employment**, Employed full or part-time %			
**Annual household income**, %			
<10,000			
10,000-19,999			
20,000-49,000			
≥50,000			
**Marital status,** Married/Engaged **%**			
**Medicaid eligible at delivery**, %			
**Access to health care**			
Having dental visit within past 2 years, %			
No insurance any time within past year, %			
**Behavior Characteristics**			
**Smoking**			
Smoking in 3 months before pregnancy, %			
Smoking during pregnancy, %			
**Drinking alcohol during pregnancy**, %			
**Pregnancy Intention**, %			
**Clinical Characteristics**			
**Gestational age at entry to study,** weeks (mean ±SD)			
**Gestational age at entry to prenatal care**, weeks (mean ±SD)			
**Parity**, Nulliparous, %			
**Previous preterm birth**, %			
**Previous prehypertension**, %			
**Vaginal infection in pregnancy**, %			
Chlamydia			
Gonorrhea			
Trichomonas			
Bacterial vaginosis			
**Any vaginal bleeding in pregnancy**, %			
**History of prior LEEP/Cervical survey**, %			
**Cerclage placed during pregnancy,** %			

**Muellerian uterine anomaly**, %	
**Cervical shortening (≤25 mm)**, %	
**Pregnancy conceived by ART**, %	
**Prepregnancy BMI**, %	
Underweight (<18.5)	
Normal (18.5-<25)	
Overweight (25-<30)	
Obese (30+)	

Figure 1: **Hypothetical** GPNC vs IPNC on **PTB** in Clinical Factor subgroups

Clinical Factors

Subgroup	GP vs IP	Adjusted OR	95% CI	p-value
**Gestational age at entry to study (weeks)**				
<25	(7.5% vs 8.2%)	0.91	(0.71, 1.16)	0.635
>=25	(8.2% vs 8.3%)	0.99	(0.77, 1.26)	
**Gestational age at entry to prenatal care (weeks)**				
<25	(7.2% vs 8.1%)	0.88	(0.69, 1.13)	0.678
>=25	(8.0% vs 8.6%)	0.92	(0.72, 1.18)	
**Parity**				
Nulliparous	(7.7% vs 8.2%)	0.93	(0.73, 1.20)	0.120
>1	(8.2% vs 8.5%)	0.96	(0.75, 1.23)	
**Previous preterm birth**				
Yes	(8.4% vs 8.5%)	0.99	(0.77, 1.26)	0.340
No	(8.0% vs 8.6%)	0.92	(0.72, 1.18)	
**Previous prehypertension**				
Yes	(8.6% vs 7.7%)	1.13	(0.88, 1.45)	0.784
No	(8.2% vs 8.5%)	0.96	(0.75, 1.23)	
**Vaginal infection in pregnancy**				
Chlamydia	(7.7% vs 8.1%)	0.95	(0.74, 1.21)	0.880
Gonorrhea	(8.0% vs 8.6%)	0.92	(0.72, 1.18)	
Trichomonas	(7.7% vs 8.1%)	0.95	(0.74, 1.21)	
Bacterial vaginosis	(8.0% vs 8.6%)	0.92	(0.72, 1.18)	
**Any vaginal bleeding in pregnancy**				
Yes	(7.7% vs 8.6%)	0.89	(0.69, 1.14)	0.554
No	(8.7% vs 8.5%)	1.03	(0.80, 1.31)	
**History of prior LEEP/Cervical survey**				
Yes	(7.5% vs 8.1%)	0.92	(0.72, 1.18)	0.111
No	(8.8% vs 8.6%)	1.03	(0.80, 1.31)	
**Cerclage placed during pregnancy**				
Yes	(7.5% vs 8.3%)	0.90	(0.70, 1.15)	0.350
No	(8.2% vs 8.4%)	0.97	(0.76, 1.25)	
**Muellerian uterine anomaly**				
Yes	(6.8% vs 8.1%)	0.83	(0.65, 1.06)	0.256
No	(9.0% vs 8.6%)	1.05	(0.82, 1.35)	
**Cervical shortening (<=25 mm)**				
Yes	(8.8% vs 8.2%)	1.08	(0.84, 1.38)	0.340
No	(8.8% vs 8.5%)	1.04	(0.81, 1.33)	
**Pregnancy conceived by ART**				
Yes	(7.7% vs 8.4%)	0.91	(0.71, 1.17)	0.790
No	(8.0% vs 8.6%)	0.92	(0.72, 1.18)	
**Prepregnancy BMI**				
Underweight (<18.5)	(7.5% vs 8.2%)	0.91	(0.71, 1.16)	0.876
Normal (18.5-<25)	(7.7% vs 8.3%)	0.92	(0.72, 1.18)	
Overweight (25-<30)	(7.9% vs 8.3%)	0.95	(0.74, 1.21)	
Obese (30+)	(8.0% vs 8.6%)	2.77	(0.72, 1.18)	

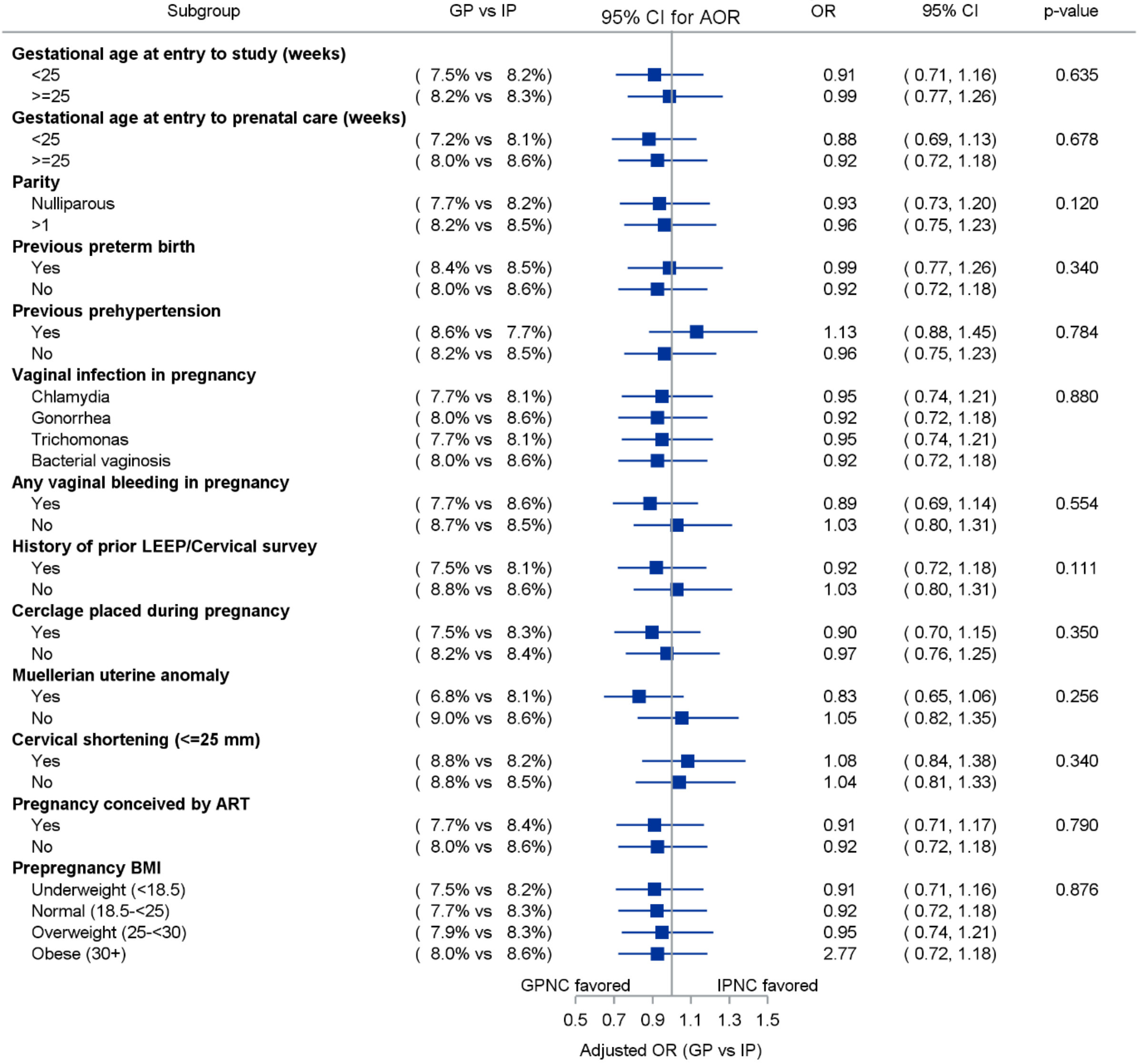

Supplement: Supplement 1. — Trial Protocol and Statistical Analysis Plan [file jamanetwopen-e2330763-s001.pdf]
