# Supplementary material for: Group vs Individual Prenatal Care and Gestational Diabetes Outcomes: A Secondary Analysis of a Randomized Clinical Trial
Source: JAMA Netw Open. 2023 Aug 29;6(8):e2330763. doi: 10.1001/jamanetworkopen.2023.30763 (PMC10466168; doi:10.1001/jamanetworkopen.2023.30763)
Supplement: Supplement 2. — eFigure. CRADLE Trial CONSORT Diagram Showing Eligibility, Randomization, Follow-Up, and Outcomes Availability, Modified Intent-to-Treat (MITT) Sample eTable 1. Baseline Characteristics and Prognostic Risk Factors for GDM by Intervention Arms: Group Prenatal Care (GPNC) Group vs. Individual Prenatal Care (IPNC) Group in CRADLE Study eTable 2. Modified Intention-to-Treat Analysis for Comparing the Risk Of GDM by Intervention Arms: Group Prenatal Care (GPNC) Group vs. Individual Prenatal Care (IPNC) Group in CRADLE Study eTable 3. Modified Intention-to-Treat Analysis for Comparing the Risks of Progression to A2 GDM and GDM-Related Obstetric Outcomes by Intervention Arms: Group Prenatal Care (GPNC) Group vs. Individual Prenatal Care (IPNC) Group in CRADLE Study eTable 4. Sensitivity Analysis for Comparing the Risk of GDM by Intervention Arms: Group Prenatal Care (GPNC) Group vs. Individual Prenatal Care (IPNC) Group in CRADLE Study eTable 5. Sensitivity Analysis for Comparing the Risks of Progression to A2 GDM and GDM-Related Obstetric Outcomes by Intervention Arms: Group Prenatal Care (GPNC) Group vs. Individual Prenatal Care (IPNC) Group in CRADLE Study [file jamanetwopen-e2330763-s002.pdf]

Supplemental Online Content

Chen Y, Crockett AH, Britt JL, et al. Group vs individual prenatal care and gestational diabetes outcomes. *JAMA Netw Open*. 2023;6(8):e2330763. doi:10.1001/jamanetworkopen.2023.30763

eFigure. CRADLE trial CONSORT Diagram Showing Eligibility, Randomization, Follow-Up, and Outcomes Availability, Modified Intent-to-Treat (MITT) Sample

eTable 1. Baseline Characteristics and Prognostic Risk Factors for GDM by Intervention Arms: Group Prenatal Care (GPNC) Group vs. Individual Prenatal Care (IPNC) Group in CRADLE Study

eTable 2. Modified Intention-to-Treat Analysis for Comparing the Risk Of GDM by Intervention Arms: Group Prenatal Care (GPNC) Group vs. Individual Prenatal Care (IPNC) Group in CRADLE Study

eTable 3. Modified Intention-to-Treat Analysis for Comparing the Risks of Progression to A2 GDM and GDM-Related Obstetric Outcomes by Intervention Arms: Group Prenatal Care (GPNC) Group vs. Individual Prenatal Care (IPNC) Group in CRADLE Study

eTable 4. Sensitivity Analysis for Comparing the Risk of GDM by Intervention Arms: Group Prenatal Care (GPNC) Group vs. Individual Prenatal Care (IPNC) Group in CRADLE Study

eTable 5. Sensitivity Analysis for Comparing the Risks of Progression to A2 GDM and GDM-Related Obstetric Outcomes by Intervention Arms: Group Prenatal Care (GPNC) Group vs. Individual Prenatal Care (IPNC) Group in CRADLE Study

This supplemental material has been provided by the authors to give readers additional information about their work.

eFigure. CRADLE trial CONSORT Diagram Showing Eligibility, Randomization, Follow-Up, and Outcomes Availability, Modified Intent-To-Treat (mITT) sample

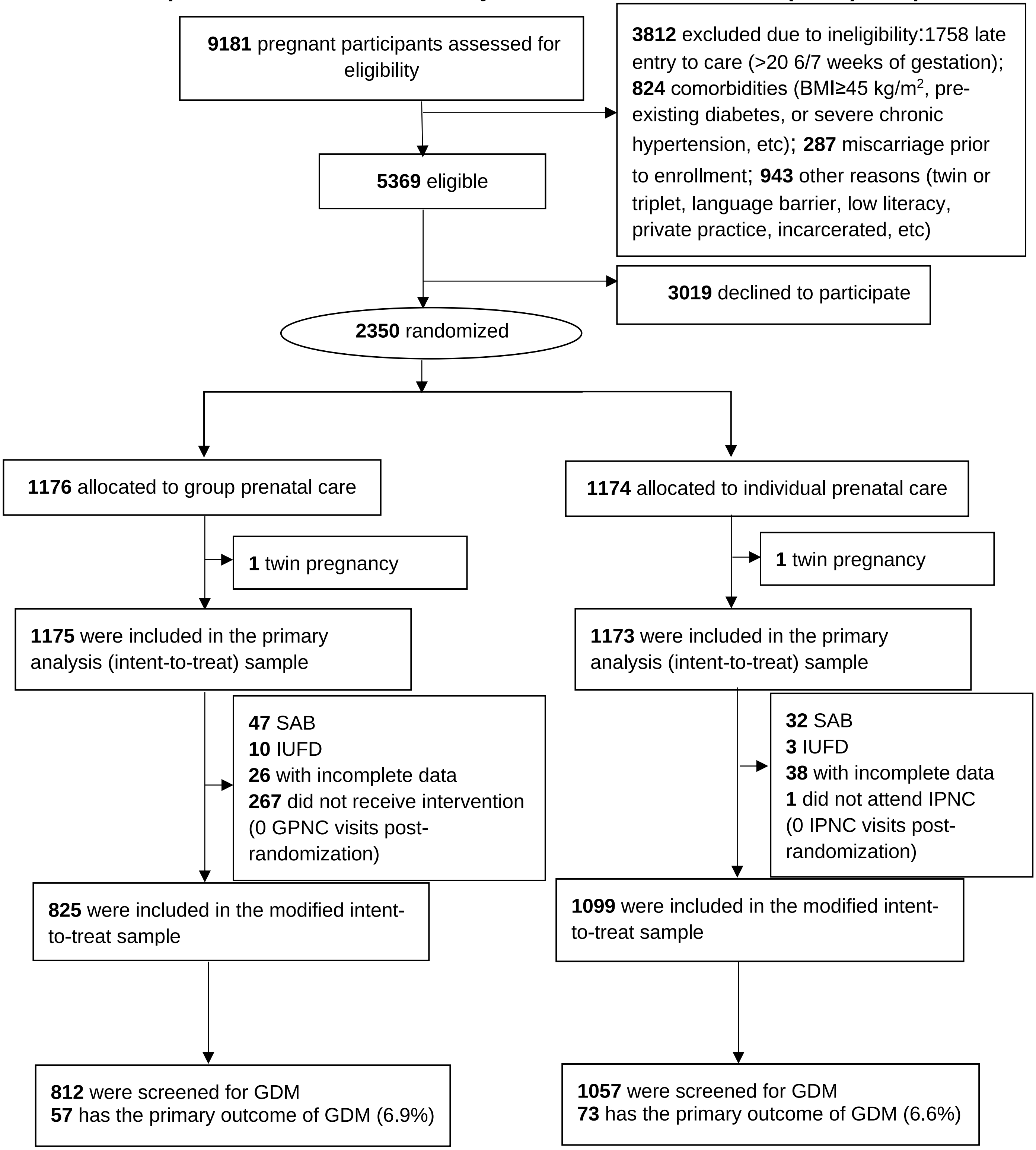

Abbreviations: BMI: Body Mass Index; GDM: Gestational Diabetes Mellitus; SAB: Spontaneous Abortion; IUFD: Intrauterine Fetal Demise; GPNC: Group Prenatal Care; IPNC: Individual Prenatal Care

© 2023 Chen Y et al. *JAMA Network Open.*

**eTable 1. Baseline Characteristics and Prognostic Risk Factors for GDM by Intervention Arms: Group Prenatal Care (GPNC) Group vs. Individual Prenatal Care (IPNC) Group in CRADLE Study[a]**		
**Characteristics, N (%)**	**IPNC Group N=1,099**	**GPNC Group N=825**
Race and ethnicity		
Black	437 (39.8)	330 (40.0)
Hispanic	238 (21.7)	193 (23.4)
White	410 (37.3)	292 (35.4)
Other/ Mixed[b]	14 (1.3)	10 (1.2)
Maternal age, Mean (SD), years	25.0 (5.3)	25.4 (5.5)
Pre-pregnancy BMI[c], Mean (SD), kg/m^2	28.8 (7.1)	28.9 (7.2)
Pre-pregnancy BMI[c] status		
Underweight (BMI < 18.5 kg/m^2)	37 (3.4)	29 (3.5)
Normal weight (18.5 ≤ BMI < 25.0 kg/m^2)	364 (33.1)	270 (32.7)
Overweight (25.0 ≤ BMI < 30.0 kg/m^2)	281 (25.6)	197 (23.9)
Obese (BMI ≥ 30.0 kg/m^2)	417 (37.9)	329 (39.9)
High school education or above	759 (69.1)	582 (70.6)
Student status for the past year		
Not a student	783 (71.3)	587 (71.2)
High school student (or working on GED)	144 (13.1)	89 (10.8)
Community, technical, and four-year college student	124 (11.3)	94 (11.4)
Employment		
Employed	554 (50.4)	432 (52.4)
Unemployed	244 (22.2)	172 (20.9)
Keeping house or caring for family full time	240 (21.8)	170 (20.6)
Marital status		
Married	701 (71.3)	537 (72.0)
Unknown	160 (14.6)	109 (13.2)
Annual household income		
Less than $10,000	237 (21.6)	183 (22.2)
$10,000 to less than $20,000	224 (20.4)	175 (21.2)
$20,000 to less than $50,000	271 (24.7)	201 (24.4)

© 2023 Chen Y et al. *JAMA Network Open.*

$50,000 or more	34 (3.1)	21 (2.6)
Unknown	333 (30.3)	245 (29.7)
Health insurance		
Had health insurance in the past year	540 (49.1)	392 (47.5)
Unknown	127 (11.6)	100 (12.1)
Nulliparous	486 (44.2)	357 (43.3)
Perceived family support, Mean (SD)	3.4 (0.6)	3.4 (0.6)
Smoked tobacco during the three months before pregnancy	425 (38.7)	247 (29.9)
Unintended pregnancy	702 (63.9)	524 (63.5)
**Prognostic factors**		
Total gestational weight gain, Mean (SD), lb	24.5 (16.6)	24.8 (16.3)
Smoked tobacco during this pregnancy	206 (18.7)	127 (15.4)
Consumed alcohol during this pregnancy	39 (3.6)	34 (4.1)
Smoked marijuana during this pregnancy	50 (4.6)	39 (4.7)

Note:

[a]The analyses were conducted among the modified intention-to-treat (mITT) sample

[b]Other indicated Asian, Native Hawaiian, Other Pacific Islander, or Unknown

[c]Pre-pregnancy BMI measured at first prenatal care visit

© 2023 Chen Y et al. *JAMA Network Open.*

eTable 2. Modified Intention-to-Treat Analysis for Comparing the Risk Of GDM by Intervention Arms: Group Prenatal Care (GPNC) Group vs. Individual Prenatal Care (IPNC) Group in CRADLE Study

	Intervention Arms		Unadjusted Risk Difference		Adjusted Risk Difference[a]	
	IPNC	GPNC	RD (95% CI)	P value	RD (95% CI)	P value
**All participants**						
GDM/Total	73/1099	57/825				
GDM incidence, %	6.6	6.9	0.27 (-2.0 to 2.5)	0.82	0.043 (-2.2 to 2.3)	0.97
**Black**						
GDM/Total	19/437	13/330				
GDM incidence, %	4.4	3.9	-0.41 (-3.3 to 2.4)	0.78	-0.42 (-3.1 to 2.3)	0.76
**Hispanic**						
GDM/Total	27/238	21/193				
GDM incidence, %	11.3	10.9	-0.46 (-6.4 to 5.5)	0.88	-1.00 (-7.2 to 5.2)	0.75
**White**						
GDM/Total	25/410	23/292				
GDM incidence, %	6.1	7.9	1.80 (-2.1 to 5.6)	0.36	1.80 (-2.1 to 5.7)	0.35

Notes:
[a]The regression model was adjusted for all baseline characteristics including smoking before pregnancy, race and ethnicity, maternal age, body mass index at enrollment, education, employment, student status for the past year, annual household income, had dental visit for the last 2 years, had insurance for the past year, parity, marital status, unintended pregnancy
Abbreviation: GDM, gestational diabetes mellitus; CI, Confidence Interval; RD, Risk Difference

© 2023 Chen Y et al. *JAMA Network Open.*

**eTable 3. Modified Intention-to-Treat Analysis for Comparing the Risks of Progression to A2 GDM and GDM-Related Obstetric Outcomes by Intervention Arms: Group Prenatal Care (GPNC) Group vs. Individual Prenatal Care (IPNC) Group in CRADLE Study**						
	**Intervention Arms**		**Unadjusted Risk Difference**		**Adjusted Risk Difference[a]**	
	**IPNC**	**GPNC**	**RD (95% CI)**	**P value**	**RD (95% CI)**	**P value**
**All participants**						
Progression to A2 GDM/Total	37/73	26/57				
Progression to A2 GDM incidence, %	50.7	45.6	-5.07 (-22.4 to 12.2)	0.57	-5.69 (-23.0 to 11.6)	0.52
Preeclampsia/Total	12/73	4/57				
Preeclampsia incidence, %	16.4	7.0	-9.42 (-20.2 to 1.4)	0.12	-6.36 (-17.4 to 4.7)	0.30
Cesarean delivery/Total	12/73	6/57				
Cesarean delivery incidence, %	16.4	10.5	-3.61 (-18.1 to 10.9)	0.63	1.70 (-12.0 to 15.4)	0.81
LGA/Total	3/73	1/57				
LGA incidence, %	4.1	1.8	-2.36 (-8.0 to 3.3)	0.46	-1.44 (-7.6 to 4.7)	0.67

Notes:
[a]The regression model was adjusted for all baseline characteristics including smoking before pregnancy, race and ethnicity, maternal age, body mass index at enrollment, education, employment, student status for the past year, annual household income, had dental visit for the last 2 years, had insurance for the past year, parity, marital status, unintended pregnancy
Abbreviation: A2 GDM, GDM managed with medication; LGA, large-for-gestational-age; CI, Confidence Interval; RD, Risk Difference

© 2023 Chen Y et al. *JAMA Network Open.*

eTable 4. Sensitivity Analysis for Comparing the Risk of GDM by Intervention Arms: Group Prenatal Care (GPNC) Group vs. Individual Prenatal Care (IPNC) Group in CRADLE Study[a]

	Intervention Arms		Unadjusted Risk Difference		Adjusted Risk Difference[b]	
	IPNC	GPNC	RD (95% CI)	P value	RD (95% CI)	P value
**All participants**						
GDM/Total	69/1087	73/1090				
GDM incidence, %	6.4	6.7	0.35 (-1.7 to 2.4)	0.74	-0.21 (-5.7 to 5.4)	0.94
**Black**						
GDM/Total	18/443	22/444				
GDM incidence, %	4.1	5.0	0.89 (-1.8 to 3.6)	0.52	0.80 (-3.2 to 4.8)	0.70
**Hispanic**						
GDM/Total	25/227	20/233				
GDM incidence, %	11.0	8.6	-2.43 (-7.9 to 3.0)	0.38	-2.73 (-8.4 to 2.9)	0.34
**White**						
GDM/Total	23/402	30/398				
GDM incidence, %	5.7	7.5	1.82 (-1.6 to 5.3)	0.30	1.25 (-9.1 to 11.6)	0.81

Notes:
[a]The analyses were conducted excluding participants whose study participation could be affected by COVID-19 pandemic.
[b]The regression model was adjusted for all baseline characteristics including smoking before pregnancy, race and ethnicity, maternal age, body mass index at enrollment, education, employment, student status for the past year, annual household income, had dental visit for the last 2 years, had insurance for the past year, parity, marital status, unintended pregnancy
Abbreviation: GDM, gestational diabetes mellitus; CI, Confidence Interval; RD, Risk Difference

© 2023 Chen Y et al. *JAMA Network Open.*

**eTable 5. Sensitivity Analysis for Comparing the Risks of Progression to A2 GDM and GDM-Related Obstetric Outcomes by Intervention Arms: Group Prenatal Care (GPNC) Group vs. Individual Prenatal Care (IPNC) Group in CRADLE Study[a]**						
	**Intervention Arms**		**Unadjusted Risk Difference**		**Adjusted Risk Difference[b]**	
	**IPNC**	**GPNC**	**RD (95% CI)**	**P value**	**RD (95% CI)**	**P value**
**All participants**						
Progression to A2 GDM/Total	36/69	34/71				
Progression to A2 GDM incidence, %	52.2	47.9	-4.29 (-20.8 to 12.3)	0.61	-6.10 (-24.5 to 12.3)	0.52
Preeclampsia/Total	11/69	6/73				
Preeclampsia incidence, %	15.9	8.2	-7.72 (-18.4 to 3.0)	0.16	-3.36 (-15.3 to 8.6)	0.58
Cesarean delivery/Total	11/69	8/73				
Cesarean delivery incidence, %	15.9	11.0	-4.98 (-16.2 to 6.2)	0.38	0.68 (-10.3 to 11.7)	0.90
LGA/Total	3/69	2/73				
LGA incidence, %	48.6	2.7	-1.61 (-7.7 to 4.5)	0.61	-0.04 (-7.4 to 7.4)	0.99

Notes:
[a]The analyses were conducted excluding participants whose study participation could be affected by COVID-19 pandemic.
[b]The regression model was adjusted for all baseline characteristics including smoking before pregnancy, race and ethnicity, maternal age, body mass index at enrollment, education, employment, student status for the past year, annual household income, had dental visit for the last 2 years, had insurance for the past year, parity, marital status, unintended pregnancy
Abbreviation: A2 GDM, GDM managed with medication; LGA, large-for-gestational-age; CI, Confidence Interval; RD, Risk Difference

© 2023 Chen Y et al. *JAMA Network Open.*
