# Supplementary material for: Group vs Individual Prenatal Care and Gestational Diabetes Outcomes: A Secondary Analysis of a Randomized Clinical Trial
Source: JAMA Netw Open. 2023 Aug 29;6(8):e2330763. doi: 10.1001/jamanetworkopen.2023.30763 (PMC10466168; doi:10.1001/jamanetworkopen.2023.30763)
Supplement: Supplement 3. — Data Sharing Statement [file jamanetwopen-e2330763-s003.pdf]

Data Sharing Statement

Chen. Group vs Individual Prenatal Care and Gestational Diabetes Outcomes. *JAMA Netw Open*. Published August 29, 2023. doi:10.1001/jamanetworkopen.2023.30763

Data
Data available: Yes
Data types: Deidentified participant data, Data dictionary, Other (please specify)
Additional Information: Deidentified study data will be available publicly on the NICHD/DASH Data and Specimen Hub (https://dash.nichd.nih.gov/) in October 2026, five years after study completion. Prior to that time, researchers with a methodologically sound proposal can direct inquiries to amy.crockett@prismahealth.org to gain access to the study protocol, informed consent forms, deidentified data, data dictionaries and the analytic plan. Requestors will need to sign a data access agreement.
How to access data: By request.
When available: beginning date: 10-01-2026

Supporting Documents
Document types: None

Additional Information
Who can access the data: Researchers whose proposed use of the data has been approved
Types of analyses: None
Mechanisms of data availability: After approval of a proposal, or with a signed data access agreement
